# Gut Microbiota Functional Traits, Blood pH, and Anti-GAD Antibodies Concur in the Clinical Characterization of T1D at Onset

**DOI:** 10.3390/ijms231810256

**Published:** 2022-09-06

**Authors:** Federica Del Chierico, Giorgia Conta, Maria Cristina Matteoli, Alessandra Fierabracci, Sofia Reddel, Gabriele Macari, Simone Gardini, Valerio Guarrasi, Stefano Levi Mortera, Valeria Marzano, Pamela Vernocchi, Fabio Sciubba, Federico Marini, Annalisa Deodati, Novella Rapini, Stefano Cianfarani, Alfredo Miccheli, Lorenza Putignani

**Affiliations:** 1 Multimodal Laboratory Medicine Research Area, Unit of Human Microbiome, Bambino Gesù Children’s Hospital, IRCCS, 00165 Rome, Italy; 2 Department of Environmental Biology, Sapienza University of Rome, 00185 Rome, Italy; 3 NMR-Based Metabolomics Laboratory of Sapienza (NMLab), Sapienza University of Rome, 00185 Rome, Italy; 4 Diabetes & Growth Disorders Unit, Bambino Gesù Children’s Hospital, IRCCS, 00165 Rome, Italy; 5 Infectivology and Clinical Trials Research Department, Bambino Gesù Children’s Hospital, IRCCS, 00165 Rome, Italy; 6 GenomeUp SRL, 00144 Rome, Italy; 7 Department of Chemistry, Sapienza University of Rome, 00185 Rome, Italy; 8 Department of Systems Medicine, University of Rome Tor Vergata, 00133 Rome, Italy; 9 Department of Women’s and Children Health, Karolisnska Institute and University Hospital, 17177 Stockholm, Sweden; 10 Department of Diagnostic and Laboratory Medicine, Unit of Microbiology and Diagnostic Immunology, Unit of Microbiomics and Multimodal Laboratory Medicine Research Area, Unit of Human Microbiome, Bambino Gesù Children’s Hospital, IRCCS, 00165 Rome, Italy

**Keywords:** gut microbiota ecology and metabolome, type 1 diabetes (T1D), pediatrics, ketoacidosis, anti-GAD antibody, insulin need, omics data integration, microbial biomarkers

## Abstract

Alterations of gut microbiota have been identified before clinical manifestation of type 1 diabetes (T1D). To identify the associations amongst gut microbiome profile, metabolism and disease markers, the 16S rRNA-based microbiota profiling and ^1^H-NMR metabolomic analysis were performed on stool samples of 52 T1D patients at onset, 17 T1D siblings and 57 healthy subjects (CTRL). Univariate, multivariate analyses and classification models were applied to clinical and -omic integrated datasets. In T1D patients and their siblings, Clostridiales and *Dorea* were increased and *Dialister* and *Akkermansia* were decreased compared to CTRL, while in T1D, Lachnospiraceae were higher and *Collinsella* was lower, compared to siblings and CTRL. Higher levels of isobutyrate, malonate, *Clostridium*, Enterobacteriaceae, Clostridiales, Bacteroidales, were associated to T1D compared to CTRL. Patients with higher anti-GAD levels showed low abundances of *Roseburia*, *Faecalibacterium* and *Alistipes* and those with normal blood pH and low serum HbA_1c_ levels showed high levels of purine and pyrimidine intermediates. We detected specific gut microbiota profiles linked to both T1D at the onset and to diabetes familiarity. The presence of specific microbial and metabolic profiles in gut linked to anti-GAD levels and to blood acidosis can be considered as predictive biomarker associated progression and severity of T1D.

## 1. Introduction

Type 1 diabetes mellitus (T1D) is an autoimmune disease caused by the progressive immunomediated destruction of pancreatic β-cells by autoreactive T lymphocytes in genetically susceptible individuals [1]. Patients with T1D develop a severe insulin deficiency that leads to hyperglycemia, requiring accurate exogenous insulin administration [2]. Markers of the β-cell destruction comprise islet cell autoantibodies (ICA), insulin autoantibodies (IAA), anti-glutamic acid decarboxylase antibodies (anti-GAD), and autoantibodies to the tyrosine phosphatases IA-2 and IA-2β [3]. However, the rate of β-cells destruction is subject-dependent, then it can be very fast in some individuals and slower in others [3]. Moreover, especially in early onset cases, some patients may show ketoacidosis as the first manifestation of the disease. Diabetic ketoacidosis (DKA) is characterized by hyperglycemia, ketosis, and metabolic acidosis [3]. During ketoacidosis, ketone bodies are produced via hepatic beta oxidation of fatty acids and released in the blood flow. This condition leads to dehydration and acidosis and it is associated with increased morbidity and mortality [4]. In general, T1D patients are characterized by a rise of inflammatory cytokines, as well as C-reactive protein (CRP) [5]. This proinflammatory condition seems to be mainly related to hyperglycemia [6]. High levels of blood glucose can activate the proinflammatory transcription factor nuclear κB (NF-κB), resulting in increased inflammatory chemokine and cytokine release [7].

In the “SEARCH for Diabetes in Youth” study, performed on 1396 young T1D patients at onset of disease, the DKA value at the diagnosis of T1D was identified as predictor of poor long-term glycemic control in children, independently of established risk factors [8]. DKA and age influence disease outcome and may thus be used to early identify those patients with rapidly deteriorating metabolic control, who might benefit from a more intensive therapeutic approach [9]. Ketoacidosis in T1D patients is indeed considered a serious complication that requires prompt intervention [10].

External factors such as life environment, diet, antibiotic exposure, and activation of the gut immune systems, have been associated with T1D pathophysiology [11,12]. Moreover, the composition of the intestinal mucus glycans can shape the gut microbiota depending on the fucosyltransferase 2 (FUT2) gene expression of the individual [13] and, indeed, FUT2 polymorphisms have been associated with several conditions, including chronic diseases and infections [14]. Recently, a possible role of the FUT2 phenotype has been suggested also in T1D pathogenesis [15].

Different studies report on an aberrant gut microbiota composition in T1D [16,17,18]. The consequence of gut dysbiosis is the impairment of gut permeability, environmentally increased exposure to non-self-antigens followed by mucosal immune response that parallel and putatively influence the development of the autoimmune attack against insulin-producing β-cell [19,20]. The modifications in gut microbiota composition and function have been identified before the β-cells destruction stage [19]. In T1D patients, gut microbiota are characterized by a reduction in: (i) bacteria producing short chain fatty acids (SCFAs) [21], (ii) fecal butyrate, (iii) intestinal immunoglobulin A (IgA) antibody levels and (iv) intestinal alkaline phosphates activity and by an increase in fecal calprotectin concentration [22]. These findings reflect the alteration of gut microbiota in T1D patients, involving butyrate and lactate producers as well as the mucin degraders [19,20].

In this study, we investigated the gut bacterial composition and its metabolic activity in an Italian cohort of children at onset of T1D, in relation to the disease clinical biomarkers at onset of disease, to identify possible disease predictors of microbial and/or metabolic nature of severity during long-term.

## 2. Results

### 2.1. The Whole Subject Cohort Characteristics

The whole cohort included 126 subjects of which 52 were patients at T1D onset (23 females [44.2%], age 9.65 ± 3.19 years), 17 T1D patients’ siblings (9 females [52.9%]), age 11.59 ± 5.24 years) and 57 healthy subjects (CTRL, 31 females [54.4%], age 9.81 ± 3.14 years). The average values of the clinical and anthropometric features for T1D patients are reported in Table 1.

### 2.2. T1D Clinical Profiling of the Overall Cohort

T1D cohort characteristics were recognized based on the cut-off values of the most representative clinical parameters for the disease (Table 1).

**Figure 1:**
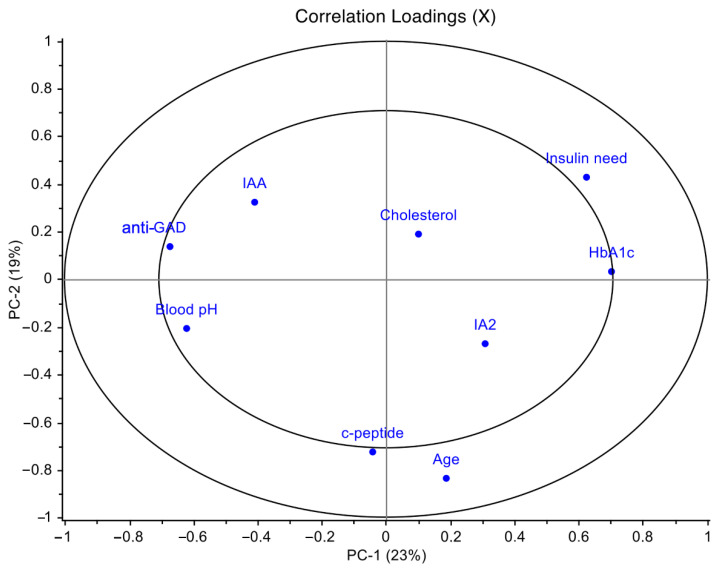
Principal component analysis (PCA) loadings plot of T1D clinical data performed on the T1D overall cohort. PCA was applied to the entire matrix of variables composed of anti-GAD, IAA, IA2, HbA_1c_, cholesterol, exogenous insulin need, blood pH, age and c-peptide, after autoscaling. These variables were selected among all the clinical parameters for T1D in order to avoid information redundancy. PCA model showed the first principal component (PC1) accounted for 23% of the overall variance and the second principal component (PC2) accounted for 19%.

PCA was applied to the matrix of variables composed of anti-GAD, IAA, IA2, HbA1c, cholesterol, exogenous insulin need, blood pH, age and c-peptide, after autoscaling. The first principal component (PC1) and the second principal component (PC2), respectively, accounted for 23% and 19% of the overall variance. The loadings plot in Figure 1 showed that the statistically significant variables insulin need and HbA_1c_ positively correlated with PC1, while anti-GAD and blood pH negatively correlated with PC1; furthermore, c-peptide and age were significant along PC2 resulting thus independent from the previous ones. 

Moreover, insulin need positively correlated with HbA_1c_ (r = 0.33; *p* = 0.03) and negatively with blood pH (r = −0.33; *p* = 0.03); anti-GAD antibodies negatively correlated with HbA_1c_ (r = −0.36; *p* = 0.02).

In the PCA scores plot, the diabetic cohort appeared to be clustered on the basis of their blood pH values. This allowed to identify the blood pH as a parameter for a further stratification of the patients (Figure S1A), linked to their severity status of the disease at the onset: patients with blood pH < 7.32, (more severe) and pH ≥ 7.32 (less severe). Moreover, higher levels of anti-GAD antibodies were mainly observed in patients with normal-to-moderate compared to low blood pH (Figure S1B).

### 2.3. Gut Microbiota Ecology of the Overall Subject Cohorts

A total of 7,916,373 sequencing reads were obtained from 126 faecal samples, with a mean value of 75,154 sequences per sample. 

**Figure 2:**
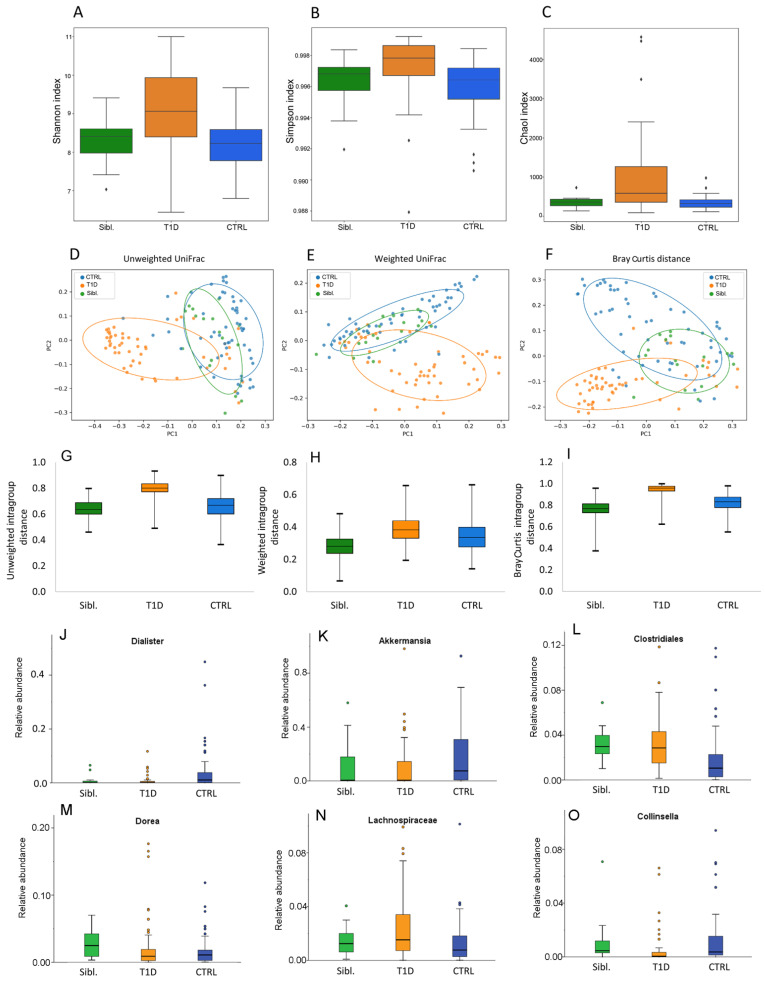
Gut microbiota ecology for siblings, T1D and CTRL groups. Panels (**A**–**C**): α-diversity Shannon, Simpson and ChaoI indexes. Panels (**D**–**F**): β-diversity PCoA plots of distance matrices calculated by unweighted UniFrac, weighted UniFrac and Bray Curtis algorithms. Panels (**G**–**I**): intragroup distance calculated by unweighted UniFrac, weighted UniFrac and Bray Curtis algorithms. Panels (**J**–**O**): Box plots showing the relative abundances of differentially abundant taxa based on a Kruskal–Wallis test (FDR *p* value < 0.1) amongst siblings (green box), T1D patients (orange box) and CTRL (blue box) groups. Box plots report median, minimum and maximum values, and the 25th and 75th percentile values of relative abundances of taxa.

Analyzing microbiota ecology amongst T1D patients, siblings and CTRL, the α-diversity indexes were higher in patients compared to the other two groups (*p* values ≤ 0.05) (Figure 2A–C).

Moreover, β-diversity showed that patient group resulted clearly separated, while CTRL and siblings resulted intermixed between each other (PERMANOVA *p* values = 0.0001) (Figure 2D–F). Moreover, based on the calculation of intragroup distance the higher distance amongst T1D samples was evident, compared to the other two groups (Figure 2G–I).

The gut microbiota composition in the three groups at phylum, family and genus levels was reported in Figure S2. The comparison among these groups showed that *Dialister* and *Akkermansia* were lower in T1D and siblings with respect to CTRL. Likewise, Clostridiales and *Dorea* were significantly more abundant in siblings and T1D than in CTRL. Higher Lachnospiraceae and lower *Collinsella* abundances were found in T1D patients than in both siblings and CTRL (Figure 2J–O) (Table S1A). However, even though *Dorea* mean abundance for T1D and siblings was significantly higher compared to CTRL, the analysis of each single couple of siblings revealed that most of them showed lower abundances compared to the CTRL’s mean (Table S1B).

### 2.4. Comparison among T1D Patients in Relation to Clinical Parameters Used for Diagnosis

The possible relationship between gut microbiota composition and clinical characteristics was investigated in patients grouped on the basis of clinical variable values, as reported in Table 1. Alpha-diversity analyses did not reveal any significant variation when the patients were grouped for all T1D parameters (Figure S3). Accordingly, also β-diversity did not reveal any statistically significant group clustering (Figure S4).

**Figure 3:**
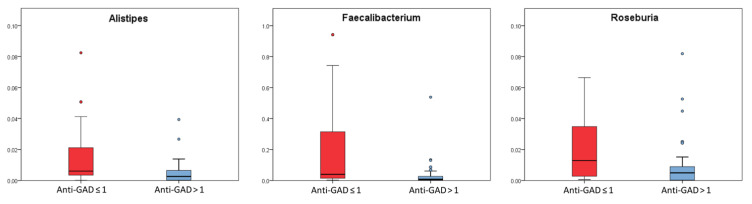
Box plots of the taxa selected basing on Kruskal–Wallis test (*p* value ≤ 0.05) for patients stratified by anti-GAD ≤ 1 (red box) and anti-GAD > 1 (light blue box). Box plots report median, minimum and maximum values, and the 25th and 75th percentile values of relative abundances of taxa.

Bacteria abundance analysis highlighted that *Alistipes*, *Roseburia* and *Faecalibacterium* abundances were significantly higher in patients with anti-GAD levels values ≤ 1 (*p* values ≤ 0.05, FDR *p* values ≤ 0.1) (Figure 3) (Table S2).

Anti-GAD antibodies negatively correlated with *Alistipes* (Pearson’s correlation test; *p* < 0.05) (Figure S5).

### 2.5. Gut Microbiota and Metabolome Profiling on the Downsized T1D and CTRL Cohorts

Metabolomics analyses were further performed on a downscaled group of patients and CTRL based on sample availability; metagenomics and metabolomics integration analyses were performed on a dataset of 43 samples and the PCA based on the clinical features was repeated (Figure S6). The obtained results were consistent with the PCA applied on the entire dataset. The PCA scores plot of T1D subset, labeled based on blood pH values, showed as well as clustering depending on blood pH values: pH < 7.32 and pH ≥ 7.32 along PC1 (Figure S7).

By ^1^H-NMR metabolomics 37 metabolites, belonging to the classes of amino acids, SCFA, organic acids, pyrimidines, purine, nitrogen compounds, carbohydrates, alcohols, were identified and quantified (Table S3).

Sensitivity and specificity of the PLS-DA model, performed on the metabolomics block only, did not reach a sufficient degree of classification for the four comparisons T1D vs. CTRL; T1D pH ≥ 7.32 vs. CTRL; T1D pH < 7.32 vs. CTRL and T1D pH ≥ 7.32 vs. T1D pH < 7.32 (Table S4).

On the contrary, the PLS-DA model performed on the metagenomics block showed % values of accuracy, sensitivity (74.0 ± 5.1) and specificity (71.2 ± 5.5) with a sufficient degree of classification for T1D vs. CTRL (Table S4).

### 2.6. Omics Data Integration

**Figure 4:**
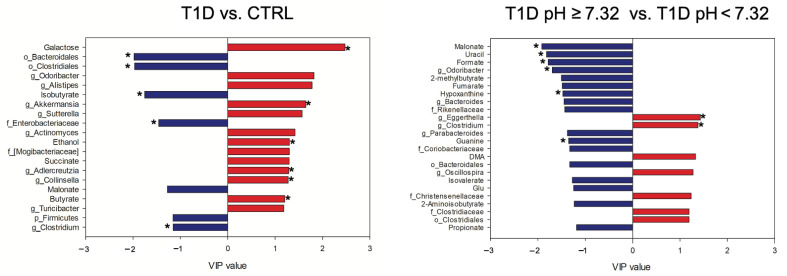
VIP values of the significant metabolites and bacterial taxa in the omics-data integration analysis. The levels of the features in blue are higher in T1D patients and in T1D pH ≥ 7.32, while the features in red are higher in CTRL and in T1D pH < 7.32. Features that resulted significative also at the univariate analysis were labeled with *. DMA, dimethylamine; Glu, glutamic acid.

Chemometric approaches were performed on the fused set, in order to try to disentangle the relationship between (i) T1D and CTRL and (ii) pH ≥ 7.32 and pH < 7.32 groups (Figure 4), as well as (iii) T1D pH ≥ 7.32 vs. CTRL and (iv) T1D pH < 7.32 vs. CTRL (Figure S8).

The PLS-DA model performed on the fused data resulted highly improved when compared to the single block analyses, as reported in Table S4. The features with values of VIP ≥1.15 were considered important and the significance was verified by performing non-parametric test with Bonferroni-corrected *p* values (Table S5). In the comparison between T1D and CTRL, T1D showed higher levels of isobutyrate, malonate, *Clostridium* (Lachnospiraceae), Enterobacteriaceae, Bacteroidales, Clostridiales and Firmicutes, while CTRL showed higher levels of butyrate, galactose, ethanol, succinate, *Odoribacter*, *Alistipes*, *Akkermansia*, *Sutterella*, *Actinomyces*, *Adlercreutzia*, *Collinsella*, *Turicibacter* and Mogibacteriaceae.

Finally, the comparison between T1D pH ≥ 7.32 and T1D pH < 7.32 revealed in T1D pH ≥ 7.32 the following features as higher: malonate, uracil, formate, 2-methylbutyrate, fumarate, hypoxanthine, guanine, isovalerate, propionate, 2-aminoisobutyrate and glutamic acid (Glu), *Odoribacter*, *Bacteroides*, *Parabacteroides*, Rikenellaceae, Coriobacteriaceae and Bacteroidales; while in T1D pH < 7.32 higher levels of dimethylamine (DMA), *Eggerthella*, *Clostridium* (Lachnospiraceae), *Oscillospira*, Christensenellaceae, Clostridiaceae and Clostridiales were reported (Figure 4).

Pearson correlations were performed on the matrix composed of both metabolites and bacterial taxa, for each considered class. Interestingly, within the group of patients with blood pH ≥ 7.32, isobutyrate was inversely correlated with *Akkermansia* (r = −0.68, *p* = 0.01). In the matrix of the CTRL, *Collinsella* was directly correlated with butyrate (r = 0.56, *p* = 0.01), succinate (r = 0.81, *p* < 0.01) and ethanol (r = 0.80, *p* < 0.01).

## 3. Discussion

The present study depicts the first integrated omics-based investigation by 16S rRNA-based metagenomics and NMR-based metabolomics, applied to a clinically uniform cohort of children affected by T1D along with their respective non-diabetic siblings and healthy reference subjects.

In our cohort of T1D patients at the onset, the α-diversity resulted increased compared to both siblings and CTRLs, as already reported [23]. Moreover, Mrozinska and colleagues proposed a positive correlation of α-diversity index and the levels of HbA_1c_ ≥ 53 mmol/mol in T1D patients [24]. This observation is consistent with our results; in fact, in our T1D cohort, HbA_1c_ values were higher than 53 mmol/mol, except one.

Comparing the gut microbiota profiles of CTRL, T1D patient and sibling groups, we observed that T1D patients and their related siblings shared higher levels of Clostridiales and lower levels of *Dialister* and *Akkermansia,* as compared to CTRL. 

The similarity in microbiota composition between T1D and siblings’ group in 14 different families, could shed a light on a phenotypic familiar predisposition that may favor a T1D characteristic gut microbiota profile. In particular, the lower presence of the beneficial *Akkermansia muciniphila*, of which increase is ever associated to a healthy intestine, could be correlated to the decrease in the intestinal integrity, and to the consequent increase in gut permeability and intestinal inflammation, that increases T1D predisposition [25].

**Figure 5:**
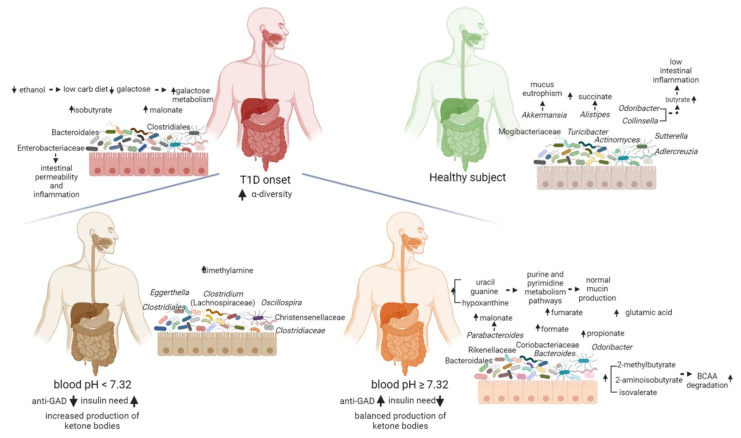
Functional descriptive model of T1D gut microbiota at onset predicting the host–microbiota interaction. Blood pH and anti-GAD antibodies resulted to be good clinical biomarkers to characterize the gut microbiota specific and functional features at the onset of T1D. T1D patients were characterized by high levels of isobutyrate, malonate, *Clostridium* (Lachnospiraceae), Enterobacteriaceae, Bacteroidales and Clostridiales unk. families, while CTRL showed higher levels of butyrate, galactose, ethanol, succinate, *Odoribacter*, *Alistipes*, *Akkermansia*, *Sutterella*, *Actinomyces*, *Adlercreutzia*, *Collinsella*, *Turicibacter* and Mogibacteriaceae. T1D pH ≥ 7.32 patients showed high values of malonate, uracil, formate, 2-methylbutyrate, fumarate, hypoxanthine, guanine, isovalerate, propionate, 2-aminoisobutyrate and glutamic acid (Glu), *Odoribacter*, *Bacteroides*, *Parabacteroides*, Rikenellaceae, Coriobacteriaceae and Bacteroidales. In T1D pH < 7.32 patients were higher levels of dimethylamine (DMA), *Eggerthella*, *Clostridium* (Lachnospiraceae), *Oscillospira*, Christensenellaceae, Clostridiaceae and Clostridiales. Created with BioRender.com.

However, T1D patients were characterized by higher levels of Lachnospiraceae and lower levels of *Collinsella*, as compared to siblings and CTRL, suggesting a specific microbiota profile associated to the diabetes at the onset. *Collinsella* is a beneficial producer of H_2_, ethanol, SCFAs, lactate and positively correlates with circulating insulin [26]. On the contrary, Lachnospiraceae have been linked to different diseases and to impaired glucose metabolism, inflammation and to the onset of T1D [20]. The unsupervised analysis (PCA) of T1D clinical data revealed that c-peptide levels were directly related to age at the time of diagnosis, and were independent from other disease severity parameters, at the onset. This analysis identified a stratification of the patients based on blood pH: pH < 7.32 (more severe) and pH ≥ 7.32 (less severe). Moreover, we observed the association between high levels of anti-GAD antibodies and normal-to-moderate pH levels and low anti-GAD antibodies levels with low blood pH values. Intriguingly, patients with low level of anti-GAD and positive to IA2 antibodies, had a more severe clinical situation compared to patients with higher anti-GAD levels that had, instead, normal blood pH values and lower insulin need (Figure 5). 

The patients with low anti-GAD levels were characterized by high abundances of *Alistipes*, *Roseburia* and *Faecalibacterium*, suggesting an association of these gut microbiota features with ketosis, and higher levels of HbA_1c_. As also reported by Fang and colleagues, the anti-GAD autoantibody strongly associates with the structure and composition of the gut microbiome but negatively correlates with SCFA-producing bacteria [27]. Our results showed that serum anti-GAD levels can be considered as a microbiota-linked predictive biomarker associated with the development of T1D. 

The integration of omics data showed an invariant core of features for the T1D children non-dependent on the severity of the disease at the onset, composed by high levels of isobutyrate, malonate, *Clostridium* (Lachnospiraceae), Enterobacteriaceae, Bacteroidales and Clostridiales unk. families, as well as low levels of butyrate, galactose, ethanol, *Alistipes, Odoribacter* and *Sutterella*. Moreover, in the Biassoni et al. study, the increase in Enterobacteriaceae and the decrease in *Sutterella* have been associated to T1D population [28].

Enterobacteriaceae overgrowth has been linked to the increase in intestinal permeability and inflammation [29]. The intestinal permeability may be considered a predisposing factor thus participating in the pathogenesis of T1D [19]. The opposite effect, i.e., the maintenance of the inter-epithelial tight junctions, is guaranteed by SCFAs, in particular butyrate, which was increased in CTRLs. Moreover, in T1D patients, a decrease in butyrogenic bacteria, i.e., *Odoribacter* and *Collinsella*, a lower butyrate production and a less butyryl-CoA transferase genes [30] was detected.

In T1D patients, malonate was increased and, in the group of patients with blood pH < 7.32, it was positively correlated with *Parabacteroides*. Interestingly, *Parabacteroides distasonis*, has been identified as the most significantly genus associated with T1D [21].

The increase in galactose metabolism pathway in T1D patients, has been already observed in a previous study [24] in which the T1D patients with HbA_1c_ ≥ 53 mmol/mol showed increased gut microbial galactose metabolism, also associated with higher α-diversity, as compared to CTRL. Moreover, an increase in the multiple sugar transport system (amongst them D-galactose) was also registered in T1D patients [20]. The passive transporting-in of nutrients is characteristic of auxotrophic bacteria that live in inflammatory environments where dead tissue provides easy access to many nutrients that are less available in the healthy gut [20].

*Alistipes* was increased in CTRL and negatively correlated with anti-GAD in T1D patients. This acetate producer seems to have a protective role for the gut health [31], despite in previous works was described as involved in inflammation processes, cancer and mental health [32].

It is noteworthy the inverse relationship between isobutyrate and butyrate in T1D patients compared to CTRL. Branched-chain fatty acids (BCFAs), such as isovalerate and isobutyrate, are produced by some gut microbes [33] responsible for the degradation pathways of leucine and valine, respectively [34]. However, isobutyrate is also produced by the activity of the B_12_-dependent Isobutyryl-CoA Mutase [35,36]. We inferred that T1D population can be characterized by bacteria with isobutyryl-CoA mutase.

Moreover, a reduced content of ethanol in stool of T1D patients, as compared to CTRL, could be related to a diet with a lower content of carbohydrates, as possibly the first tentative of nutritional intervention prior to the clinical diagnosis. 

Finally, by comparing diabetic patients on the basis of their differences in blood pH, we obtained lower classification values compared to T1D vs. CTRL results. Despite the loss of this statistical power, this model could be considered noteworthy since still allowed us to discriminate patients who shared the same disease. Intriguingly, the features that discriminate T1D patients are mostly metabolites, suggesting a major role of the metabolism in T1D progression. In particular, T1D patients characterized by blood pH ≥ 7.32, showed high level of uracil, formate, guanine and hypoxanthine. The increase in guanine and hypoxanthine led us to assume the possible involvement of purine/pyrimidine metabolism pathways in the disease progression. The gut microbiota releases purine compounds available to the intestinal mucosa that can be therefore used for nucleotide genesis [37]. We inferred that, when patients have still a balanced production of ketone bodies, the described mechanism occurs maintaining the integrity of the mucosal barrier and guaranteeing a normal mucin intestinal production. In fact, it is known that an undamaged gut barrier can shield against the entry of infectious agents and dietary antigens, avoiding immune reactions with damage to pancreatic β-cells, increased cytokine levels and insulin resistance [38].

There are several limitations of the study. First, it still requires further clinical studies with a larger sample size to validate the functional and compositional fecal microbial profiles associate to the progression and severity of T1D. Second, animal experiments could help to determine the cause-effect relationship amongst gut microbiota composition and the destruction of pancreatic β-cells. Finally, for future longitudinal studies, the exploration of the alterations or restoration of the fecal microbiota after insulin treatment could be take into consideration.

## 4. Materials and Methods

### 4.1. Patient Recruitment

A cohort of 52 consecutive Caucasian children were enrolled within one week of T1D diagnosis at the Endocrinology and Diabetes Unit of Children’s Hospital Bambino Gesù in Rome, Italy. Inclusion criteria were age between 5 and 15 years, glycemia > 126 mg/dL, glycated hemoglobin (HbA_1c_) > 6.5% (48 mmol/mol), c-peptide < 1 ng/mL. Exclusion criteria were other chronic and infective disease; use of antibiotic, pre/probiotic and propton-pump inhibitors in the last two months from recruitment. 

Age at onset, gender, weight, birth weight, pubertal stage, blood pH, exogenous insulin need, body mass index (BMI), IAA, islet antigen 2 antibody (IA-2), anti-GAD, HbA_1c_, High Density Lipoprotein Cholesterol (HDL), Low Density Lipoprotein Cholesterol (LDL), C-Reactive Protein (CRP) and presence of other autoimmune diseases (i.e., thyroiditis and celiac disease) were registered for each patient at the time of enrollment. 

A cohort of 17 children that had a full sibling diagnosed with T1D was recruited at the Endocrinology and Diabetes Unit of Children’s Hospital Bambino Gesù in Rome, Italy. Inclusion criteria were age between 5 and 17 years, general healthy condition, no gastrointestinal (GI) diseases, use of antibiotic, pre/probiotic in the previous two months from recruitment. 

A cohort of 57 gender- and age-matched controls were enrolled during an epidemiological survey carried out at the Human Microbiome Unit of Bambino Gesù Children’s Hospital in Rome (BBMRI Human Micro-biome Biobank, OPBG) to generate a reference digital biobank of healthy subjects (CTRL). This cohort did not have family history for autoimmune diseases. Moreover, they were normal weight and had no GI diseases, use of antibiotic, and pre/probiotic in the previous two months before the recruitment. 

All patients, siblings and CTRLs followed a Mediterranean diet regimen attested by Food Frequency questionnaire (FFQ) to assess the adherence to the Mediterranean diet through targeted questions about the frequency of consumption of extra virgin olive oil, butter or margarine, fresh fruit and vegetables, legumes, fish, red meat and sausages, white meat, industrial bakery products, dried fruit sugary drinks and alcohol use.

The study was approved by the OPBG Ethical Committee (protocol 1274_OPBG_2016; healthy subjects: Protocol No. 1113_OPBG_2016) and was conducted in accordance with the Principles of Good Clinical Practice and the Declaration of Helsinki. Written informed consent was obtained from either parents or legal representative of children. 

**Figure 6:**
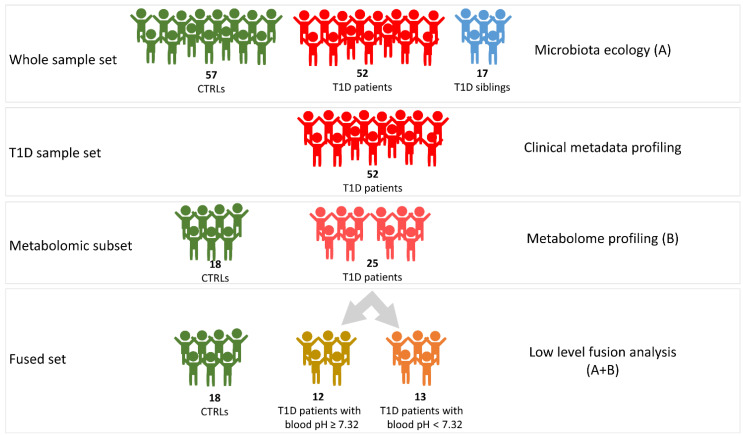
Graphic representation of the workflow of omics analyses. A, microbiota ecology outcome; B, metabolome profiling outcome. A + B, integration of metabolomic and metagenomic results.

From each subject of these cohorts, a single fecal sample was collected and stored at −80 °C until further analyses. The T1D overall set was analyzed for gut microbiota ecology by 16S rRNA-based metagenomics. For the metabolomics and, as well, for the multi-block approach, analyses were performed on a sub-set of samples (“fused set”), consistently with material availability (Figure 6).

### 4.2. Biochemical and Immunological Analyses

Glycated hemoglobin (HbA1c) was analyzed by a spectrophotometric method (Bio-Rad Richmond, CA, USA). C-peptide were measured by chemiluminescence on an ADVIA Centaur^®^ XPT Immunoassay System (Siemens Healthcare GmbH, Munich, Germany), a two-site sandwich immunoassay using direct chemiluminescent technology. Total cholesterol, LDL and HDL cholesterol, and triglycerides were measured by an enzymatic method on a Roche automated clinical chemistry analyzer (Roche/Hitachi 904 analyzer, Roche Diagnostics, Mannheim, Germany). Anti-GAD, IA2, IAA antibodies were measured by Elisa method on Elite Impatto Twin plus (EUROSPITAL diagnostics, Trieste, Italy).

### 4.3. 16S rRNA-Based Microbiota Profiling

DNA was extracted from 200 mg of stools using QIAmp Fast DNA Stool mini kit (Qiagen, Germany), following the manufacturer’s instructions. The 16S rRNA V3-V4 variable region (~460 bp) was amplified by using the primer pairs described in the MiSeq rRNA Amplicon Sequencing protocol (Illumina, San Diego, CA, USA). The PCR reactions were set up using a 2× KAPA Hifi HotStart ready Mix (KAPA Biosystems Inc., Wilmington, MA, USA) following the manufacturer’s protocol. AMPure XP beads (Beckman Coulter Inc., Beverly, MA, USA) were employed to clean DNA amplicons from primers and dimer primers. A unique combination of Illumina Nextera adaptor-primers for each sample was incorporated in amplicons by a second amplification step. The final library was cleaned-up and quantified using Quant-iT™ PicoGreen^®^ dsDNA Assay Kit (Thermo Fisher Scientific, Waltham, MA, USA). Samples were pooled together before the sequencing on an Illumina MiSeqTM platform according to the manufacturer’s specifications to generate paired-end reads of 300 base-length [39]. The bioinformatic data analysis was performed using the QIIME 2 pipeline [40]. Forward and reverse raw fastq files were merged using PEAR v. 0.9.6. Merged reads were filtered for chimeras and length using DADA2 plugin of QIIME2 [41] with a trunc value of 400 nucleotides (nt). Taxonomy was assigned against the Greengenes 13_08 database, using a Naïve bayes classifier trained on the reference sequences of Greengenes 13_08 clustered at 99% of sequence similarity [42]. Data normalization was performed in R 4.0.3 while data filtering was performed by using custom scripts in python 3.6. The read number count was normalized using Cumulative Sum Scaling (CSS) method as implemented in the MetagenomeSeq (version 3.12) package of R. Clusters (Amplicon sequence variants [ASVs]) with a number of reads lower than 1% of the total read number were removed from the statistical analysis together with those taxa not present in at least 25% of the samples [43]. α-diversity was performed by scikit-bio (http://scikit-bio.org/, accessed on 31 March 2022) of Python 3 package and the *p* value for group comparisons was determined by Kruskal–Wallis test. β-diversity analysis was calculated on weighted and unweighted Unifrac distance matrices and Bray Curtis matrix, and graphed by principal coordinate analysis (PCoA) plots. The association between the covariates and β-diversity measures was assessed by permutational analysis of variance (PERMANOVA) [44]. The non-parametric Kruskal–Wallis test, corrected for FDR *p* value ≤ 0.05, was used to compare bacterial taxa relative abundance amongst groups. The correlation between clinical variables and bacterial taxa was calculated by Pearson’s coefficient with relative *p* value. Ternary scatter plot on bacterial taxa relative abundances at phylum, family and genus levels was performed by Plotly Express of python.

### 4.4. ^1^H-NMR Metabolomic Analysis

An average quantity of 300 mg of frozen stools recovered from each sample was combined with 1 mL of PBS-D_2_O with 0.3% (final concentration) of sodium azide. The samples were thawed for 30 min at 25 °C and then vortexed to achieve a homogenous solution. The supernatant was separated from the solid phase through a first centrifugation at 10,000× *g* for 25 min at 4 °C, hence filtered on a 40 μm pores filter. After adding 200 μL of PBS-D_2_O with 0.3% of sodium azide, the samples were centrifuged again at 10,000× *g* for 25 min at 4 °C. Six hundred μL of supernatant were withdrawn and 60 μL of PBS-D_2_O containing 3-(trimethylsilyl) propionic-2,2,3,3-d_4_ acid sodium salt (TSP, 2mM final concentration) were added. NMR spectra were acquired using a Bruker Avance III 400 spectrometer (Bruker BioSpin GmbH, Karlsruhe, Germany) equipped with a 9.4T magnet operating at ^1^H frequency of 400.13MHz and at 298K. The assignment was achieved by means of bi-dimensional (2D) experiments (COSY, TOCSY, HSQC, HMBC) on selected samples and confirmed by comparison with web database [45], the literature [46,47,48,49] and *in-house* database. One-dimensional (1D) NMR spectra were processed and quantified by using ACD/Lab 1D NMR Manager ver. 12.0 software (Advanced Chemistry Development, Inc., Toronto, ON, Canada), whereas 2D-NMR spectra were processed by using Bruker TopSpin ver.3.1 (Bruker BioSpin GmbH) and MestreC ver.4.7.0.0 (Mestrelab Research SL, Santiago de Compostela, Spain). Phase and baseline of the NMR spectra were manually corrected. The quantification was carried out by comparing the integrals of the metabolites’ resonances to the TSP one, then normalizing for the number of protons and for feces weight.

### 4.5. Statistical Analyses and Modeling

To evaluate the differences in stool metabolic profiles among T1D patients and CTRL, as well as among severity status subgroups, were performed multivariate and univariate analyses. As the first step, an unsupervised approach, by means of Principal Component Analysis (PCA), was applied to the matrix of the clinical variables composed of anti-GAD, IAA, IA2, HbA1c, cholesterol, exogenous insulin need, blood pH, age and c-peptide (Figure S2) in order to highlight possible patients’ clusters, to identify outliers and features of interest. All data were autoscaled before further data processing: operationally, each variable was first centered by subtracting its average from the data and then scaled through division by its standard deviation. The scores plot allowed to identify the blood pH as a parameter for the further stratification of the patients based on the severity status of the disease at the onset: T1D patients with blood pH ≥ 7.32 and pH < 7.32 (Figure S3B). To evaluate the contribution of the metabolites and bacterial taxa for each class that were identified, a classification strategy based on the Partial Least Squares-Discriminant Analysis (PLS-DA) algorithm was applied on both metabolomics and the metagenomics data sets (Table S4). In this context, to evaluate the reliability of the prediction models and to identify a set of features significantly (and consistently) contributing to the model, an approach based on repeated double cross-validation (rDCV) was adopted.

### 4.6. Multi-Omics Data Integration

Since data were obtained by NMR-based metabolomics and taxonomical metagenomics, a multi-block (low-level fusion) approach was followed [50] to extract, simultaneously, the maximum of the information from the two approaches. Indeed, rather than being separately processed, the matrices were jointly elaborated to highlight the correlations between metabolites and bacterial taxa. For the classification stage, the predictive analyses were carried out by means of a multi-block partial least squares discriminant analysis (MB-PLSDA). MB-PLSDA [50] consists in building a PLS-DA model on the concatenated matrix resulting from the low-level data fusion of the two different blocks, after scaling each one through division by its Frobenius’ norm in order to level out their relative contribution [50]. In particular, defining ***X****_mb_* and ***X****_mg_* the matrices resulting from the metabolomic and metagenomic analysis, respectively, their squared Frobenius’ norm is given by the sum of their squared elements:

(1) $$\|X_{i}\|{}_{F}^{2}=\sum_{j,k}{\left(x_{jk}\right)}_{i}^{2}$$

where $X_{i}$ is either $X_{mb}$ or $X_{mg}$ and ${\left(x_{jk}\right)}_{i}$ is the generic element of that matrix. In order to perform multi-block modelling, $X_{mb}$ or $X_{mg}$ are normalized and concatenated row-wise (low-level fusion) to obtain the matrix $X_{conc}$:

(2) $$X_{conc}=\left[\begin{array}{cc} \frac{X_{mb}}{\|X_{mb}\|{}_{F}} & \frac{X_{mg}}{\|X_{mg}\|{}_{F}} \end{array}\right]$$

Standard PLS-DA is then used to build the classification model based on $X_{conc}$.

Since MB-PLSDA is a predictive model, a validation phase is needed to evaluate the reliability of its prediction. To this purpose, an approach based on repeated double cross-validation (rDCV) strategy, to estimate the confidence intervals for the model predictions and the consistence of candidate biomarkers based on their VIP value, was applied. Finally, to rule out any possibility that good classification results could be obtained by chance, permutation tests were applied to estimate in a non-parametrical fashion the distributions of the classification figures of merit under the null hypothesis for significance testing.

The predictive models have been applied to the comparisons: (i) T1D vs. CTRL; (ii) pH < 7.32 vs. CTRL; (iii) pH ≥ 7.32 vs. CTRL; and (iv) pH ≥ 7.32 vs. pH < 7.32 groups.

Following the multivariate results, to the significant metabolites and bacterial taxa Mann–Whitney’s *u*-test was applied to assess differences in the levels within each of the specific class. Bonferroni-corrected *p* values ≤ 0.05 were considered significant.

### 4.7. Data and Resource Availability

All raw sequences have been archived in the NCBI database: PRJNA702261 and PRJNA280490 (https://www.ncbi.nlm.nih.gov/bioproject).

## 5. Conclusions

Our results evidenced both specific gut microbiota profiles in T1D patients at the onset and other linked to familiarity. Thus, a comprehensive characterization of the gut microbiota could be associated to a genetic test to define the diabetes predisposition in a T1D familial context. Moreover, the presence of specific microbial taxa in gut linked to the serum anti-GAD levels can be considered as a microbiota predictive biomarker associated with the progression of T1D. Furthermore, we find gut microbiota specific functional traits associated to blood acidosis, indicating a role of gut microbiota in disease severity. Therefore, the routine characterization of the composition and function of the gut microbiota could be useful in patients’ clinical monitoring to assess disease status and progression. This evidence could open new avenues on patient’s treatments at onset, exploring the opportunity to combine insulin administration with probiotics, prebiotics or fecal microbiota transplantations (FMT) at onset and during clinical disease progression.

## Figures and Tables

**Table 1:** Clinical and anthropometric parameters of T1D patients, siblings and CTRLs.

Parameter		T1D Average ± SD	Siblings Average ± SD	CTRLs Average ± SD
Birth weight (kg)		3.24 ± 0.55	3.02 ± 0.55	3.32 ± 0.42
BMI (kg/m^2^)		16.48 ± 2.84	19.88 ± 2.09	17.61 ± 1.61
Total Cholesterol (mg/dL)		155.65 ± 49.87	152.00 ± 14.76	153.98 ± 16.24
HDL-C (mg/dL)		42.44 ± 13.30	49.59 ± 3.12	50.39 ± 4.16
LDL-C (mg/dL)		95.70 ± 33.03	91.47 ± 3.12	94.81 ± 6.51
Triglycerides (mg/dL)		91.79 ± 45.65	60.47 ± 6.18	59.02 ± 6.32
Pubertal stage (PS)	PS = 1	28 (53.8)	5 (29.4)	31 (54.4)
	PS = 2–3	13 (25)	4 (23.5)	18 (31.6)
	PS = 4–5	11 (21.2)	8 (47.1)	8 (14.0)
**T1D Patients Features**				
**Parameter**		**Average ± SD**	**Variable Cut Off**	**Cases N (%)**
Age at onset (years)		9.52 ± 3.21	Age at onset > 14 years	6 (11.5)
Blood pH at onset		7.26 ± 0.12	normal pH ≥ 7.32	24 (46.1)
			moderate 7.10 ≤ pH < 7.32	24 (46.1)
			severe pH < 7.10	4 (7.6)
Exogenous insulin need (IU/kg BM)		0.84 ± 0.25	Exogenous insulin need ≥1 IU/kg BM	15 (28.8)
IAA (U/mL)		6.38 ± 3.56	IAA ≥ 7 U/mL	18 (53.8)
IA-2 (U/mL)		40.56 ± 208.44	IA-2 > 1 U/mL	42 (80.8)
Anti-GAD (U/mL)		31.80 ± 40.91	Anti-GAD > 1 U/mL	31 (59.6)
c-peptide (ng/mL)		0.31 ± 0.19	c-peptide < 1 ng/mL	52 (100)
HbA_1c_ (%)		102.37 ± 22.71	HbA_1c_ > 48 mmol/mol	52 (100)
CRP (mg/L)		0.45 ± 1.40	CRP > 1 mg/L	4 (7.6)
Presence of other autoimmune diseases (i.e., Thyroiditis and celiac disease)				9 (17.3)

SD, standard deviation; BMI, body mass index; IAA, insulin autoantibodies; IA-2, islet antigen 2 antibody; anti-GAD, anti-glutamic acid decarboxylase antibody; HbA1c, glycated hemoglobin; HDL, High Density Lipoprotein Cholesterol; LDL, Low Density Lipoprotein Cholesterol; CRP, C-Reactive Protein.

## Data Availability

All raw sequences have been archived in NCBI database: PRJNA702261 and PRJNA280490 (https://www.ncbi.nlm.nih.gov/bioproject, accessed on 31 March 2022).

## Supplementary Materials

The supporting information can be downloaded at: https://www.mdpi.com/article/10.3390/ijms231810256/s1.

## References

[1] Atkinson M.A., Eisenbarth G.S., Michels A.W. (2014). Type 1 Diabetes. Lancet.

[2] American Diabetes Association (2013). Standards of Medical Care in Diabetes—2013. Diabetes Care.

[3] (2010). American Diabetes Association Diagnosis and Classification of Diabetes Mellitus. Diabetes Care.

[4] Castellanos L., Tuffaha M., Koren D., Levitsky L.L. (2020). Management of Diabetic Ketoacidosis in Children and Adolescents with Type 1 Diabetes Mellitus. Pediatr. Drugs.

[5] Stentz F.B., Umpierrez G.E., Cuervo R., Kitabchi A.E. (2004). Proinflammatory Cytokines, Markers of Cardiovascular Risks, Oxidative Stress, and Lipid Peroxidation in Patients With Hyperglycemic Crises. Diabetes.

[6] Doi Y., Kiyohara Y., Kubo M., Tanizaki Y., Okubo K., Ninomiya T., Iwase M., Iida M. (2005). Relationship Between C-Reactive Protein and Glucose Levels in Community-Dwelling Subjects without Diabetes: The Hisayama Study. Diabetes Care.

[7] Shanmugam N., Reddy M.A., Guha M., Natarajan R. (2003). High Glucose-Induced Expression of Proinflammatory Cytokine and Chemokine Genes in Monocytic Cells. Diabetes.

[8] Duca L.M., Reboussin B.A., Pihoker C., Imperatore G., Saydah S., Mayer-Davis E., Rewers A., Dabelea D. (2019). Diabetic Ketoacidosis at Diagnosis of Type 1 Diabetes and Glycemic Control over Time: The SEARCH for Diabetes in Youth Study. Pediatr Diabetes.

[9] Duca L.M., Wang B., Rewers M., Rewers A. (2017). Diabetic Ketoacidosis at Diagnosis of Type 1 Diabetes Predicts Poor Long-Term Glycemic Control. Diabetes Care.

[10] Modi A., Agrawal A., Morgan F. (2017). Euglycemic Diabetic Ketoacidosis: A Review. Curr Diabetes Rev.

[11] Davis-Richardson A.G., Triplett E.W. (2015). A Model for the Role of Gut Bacteria in the Development of Autoimmunity for Type 1 Diabetes. Diabetologia.

[12] Livanos A.E., Greiner T.U., Vangay P., Pathmasiri W., Stewart D., McRitchie S., Li H., Chung J., Sohn J., Kim S. (2016). Antibiotic-Mediated Gut Microbiome Perturbation Accelerates Development of Type 1 Diabetes in Mice. Nat. Microbiol..

[13] Kashyap P.C., Marcobal A., Ursell L.K., Smits S.A., Sonnenburg E.D., Costello E.K., Higginbottom S.K., Domino S.E., Holmes S.P., Relman D.A. (2013). Genetically Dictated Change in Host Mucus Carbohydrate Landscape Exerts a Diet-Dependent Effect on the Gut Microbiota. Proc. Natl. Acad. Sci. USA.

[14] Smyth D.J., Cooper J.D., Howson J.M.M., Clarke P., Downes K., Mistry T., Stevens H., Walker N.M., Todd J.A. (2011). *FUT2* Nonsecretor Status Links Type 1 Diabetes Susceptibility and Resistance to Infection. Diabetes.

[15] Giampaoli O., Conta G., Calvani R., Miccheli A. (2020). Can the FUT2 Non-Secretor Phenotype Associated With Gut Microbiota Increase the Children Susceptibility for Type 1 Diabetes? A Mini Review. Front. Nutr..

[16] Endesfelder D., zu Castell W., Ardissone A., Davis-Richardson A.G., Achenbach P., Hagen M., Pflueger M., Gano K.A., Fagen J.R., Drew J.C. (2014). Compromised Gut Microbiota Networks in Children With Anti-Islet Cell Autoimmunity. Diabetes.

[17] de Goffau M.C., Fuentes S., van den Bogert B., Honkanen H., de Vos W.M., Welling G.W., Hyöty H., Harmsen H.J.M. (2014). Aberrant Gut Microbiota Composition at the Onset of Type 1 Diabetes in Young Children. Diabetologia.

[18] Murri M., Leiva I., Gomez-Zumaquero J.M., Tinahones F.J., Cardona F., Soriguer F., Queipo-Ortuño M.I. (2013). Gut Microbiota in Children with Type 1 Diabetes Differs from That in Healthy Children: A Case-Control Study. BMC Med..

[19] Brown C.T., Davis-Richardson A.G., Giongo A., Gano K.A., Crabb D.B., Mukherjee N., Casella G., Drew J.C., Ilonen J., Knip M. (2011). Gut Microbiome Metagenomics Analysis Suggests a Functional Model for the Development of Autoimmunity for Type 1 Diabetes. PLoS ONE.

[20] Kostic A.D., Gevers D., Siljander H., Vatanen T., Hyötyläinen T., Hämäläinen A.-M., Peet A., Tillmann V., Pöhö P., Mattila I. (2015). The Dynamics of the Human Infant Gut Microbiome in Development and in Progression toward Type 1 Diabetes. Cell Host Microbe.

[21] Vatanen T., Franzosa E.A., Schwager R., Tripathi S., Arthur T.D., Vehik K., Lernmark Å., Hagopian W.A., Rewers M.J., She J.-X. (2018). The Human Gut Microbiome in Early-Onset Type 1 Diabetes from the TEDDY Study. Nature.

[22] Lassenius M.I., Fogarty C.L., Blaut M., Haimila K., Riittinen L., Paju A., Kirveskari J., Järvelä J., Ahola A.J., Gordin D. (2017). Intestinal Alkaline Phosphatase at the Crossroad of Intestinal Health and Disease—A Putative Role in Type 1 Diabetes. J Intern Med.

[23] Liu X., Cheng Y.-W., Shao L., Sun S.-H., Wu J., Song Q.-H., Zou H.-S., Ling Z.-X. (2021). Gut Microbiota Dysbiosis in Chinese Children with Type 1 Diabetes Mellitus: An Observational Study. World J. Gastroenterol..

[24] Mrozinska S., Kapusta P., Gosiewski T., Sroka-Oleksiak A., Ludwig-Słomczyńska A.H., Matejko B., Kiec-Wilk B., Bulanda M., Malecki M.T., Wolkow P.P. (2021). The Gut Microbiota Profile According to Glycemic Control in Type 1 Diabetes Patients Treated with Personal Insulin Pumps. Microorganisms.

[25] Stewart C.J., Ajami N.J., O’Brien J.L., Hutchinson D.S., Smith D.P., Wong M.C., Ross M.C., Lloyd R.E., Doddapaneni H., Metcalf G.A. (2018). Temporal Development of the Gut Microbiome in Early Childhood from the TEDDY Study. Nature.

[26] Gomez-Arango L.F., Barrett H.L., Wilkinson S.A., Callaway L.K., McIntyre H.D., Morrison M., Dekker Nitert M. (2018). Low Dietary Fiber Intake Increases Collinsella Abundance in the Gut Microbiota of Overweight and Obese Pregnant Women. Gut Microbes.

[27] Fang Y., Zhang C., Shi H., Wei W., Shang J., Zheng R., Yu L., Wang P., Yang J., Deng X. (2021). Characteristics of the Gut Microbiota and Metabolism in Patients With Latent Autoimmune Diabetes in Adults: A Case-Control Study. Diabetes Care.

[28] Biassoni R., Di Marco E., Squillario M., Barla A., Piccolo G., Ugolotti E., Gatti C., Minuto N., Patti G., Maghnie M. (2020). Gut Microbiota in T1DM-Onset Pediatric Patients: Machine-Learning Algorithms to Classify Microorganisms as Disease Linked. J. Clin. Endocrinol. Metab..

[29] Baldelli V., Scaldaferri F., Putignani L., Del Chierico F. (2021). The Role of Enterobacteriaceae in Gut Microbiota Dysbiosis in Inflammatory Bowel Diseases. Microorganisms.

[30] Elhag D.A., Kumar M., Al Khodor S. (2020). Exploring the Triple Interaction between the Host Genome, the Epigenome, and the Gut Microbiome in Type 1 Diabetes. Int. J. Mol. Sci..

[31] Oliphant K., Allen-Vercoe E. (2019). Macronutrient Metabolism by the Human Gut Microbiome: Major Fermentation by-Products and Their Impact on Host Health. Microbiome.

[32] Parker B.J., Wearsch P.A., Veloo A.C.M., Rodriguez-Palacios A. (2020). The Genus Alistipes: Gut Bacteria With Emerging Implications to Inflammation, Cancer, and Mental Health. Front. Immunol..

[33] Huang S., Kleerebezem R., Rabaey K., Ganigué R. (2020). Open Microbiome Dominated by Clostridium and Eubacterium Converts Methanol into I-Butyrate and n-Butyrate. Appl. Microbiol. Biotechnol..

[34] Manoli I., Venditti C.P. (2016). Disorders of Branched Chain Amino Acid Metabolism. Transl. Sci. Rare Dis..

[35] Cracan V., Banerjee R. (2012). Novel B _12_ -Dependent Acyl-CoA Mutases and Their Biotechnological Potential. Biochemistry.

[36] Jost M., Born D.A., Cracan V., Banerjee R., Drennan C.L. (2015). Structural Basis for Substrate Specificity in Adenosylcobalamin-Dependent Isobutyryl-CoA Mutase and Related Acyl-CoA Mutases. J. Biol. Chem..

[37] Lee J.S., Wang R.X., Goldberg M.S., Clifford G.P., Kao D.J., Colgan S.P. (2020). Microbiota-Sourced Purines Support Wound Healing and Mucous Barrier Function. iScience.

[38] de Kort S., Keszthelyi D., Masclee A.A.M. (2011). Leaky Gut and Diabetes Mellitus: What Is the Link?: Leaky Gut in Diabetes. Obes. Rev..

[39] Romani L., Del Chierico F., Chiriaco M., Foligno S., Reddel S., Salvatori G., Cifaldi C., Faraci S., Finocchi A., Rossi P. (2020). Gut Mucosal and Fecal Microbiota Profiling Combined to Intestinal Immune System in Neonates Affected by Intestinal Ischemic Injuries. Front. Cell Infect Microbiol..

[40] Bolyen E., Rideout J.R., Dillon M.R., Bokulich N.A., Abnet C.C., Al-Ghalith G.A., Alexander H., Alm E.J., Arumugam M., Asnicar F. (2019). Reproducible, Interactive, Scalable and Extensible Microbiome Data Science Using QIIME 2. Nat. Biotechnol..

[41] Callahan B.J., McMurdie P.J., Rosen M.J., Han A.W., Johnson A.J.A., Holmes S.P. (2016). DADA2: High-Resolution Sample Inference from Illumina Amplicon Data. Nat Methods.

[42] Bokulich N.A., Kaehler B.D., Rideout J.R., Dillon M., Bolyen E., Knight R., Huttley G.A., Gregory Caporaso J. (2018). Optimizing Taxonomic Classification of Marker-Gene Amplicon Sequences with QIIME 2’s Q2-Feature-Classifier Plugin. Microbiome.

[43] Bokulich N.A., Subramanian S., Faith J.J., Gevers D., Gordon J.I., Knight R., Mills D.A., Caporaso J.G. (2013). Quality-Filtering Vastly Improves Diversity Estimates from Illumina Amplicon Sequencing. Nat. Methods.

[44] Chen J., Bittinger K., Charlson E.S., Hoffmann C., Lewis J., Wu G.D., Collman R.G., Bushman F.D., Li H. (2012). Associating Microbiome Composition with Environmental Covariates Using Generalized UniFrac Distances. Bioinformatics.

[45] Wishart D.S., Jewison T., Guo A.C., Wilson M., Knox C., Liu Y., Djoumbou Y., Mandal R., Aziat F., Dong E. (2012). HMDB 3.0—The Human Metabolome Database in 2013. Nucleic Acids Res..

[46] Jacobs D.M., Deltimple N., van Velzen E., van Dorsten F.A., Bingham M., Vaughan E.E., van Duynhoven J. (2008). ^1^ H NMR Metabolite Profiling of Feces as a Tool to Assess the Impact of Nutrition on the Human Microbiome. NMR Biomed..

[47] Praticò G., Capuani G., Tomassini A., Baldassarre M.E., Delfini M., Miccheli A. (2014). Exploring Human Breast Milk Composition by NMR-Based Metabolomics. Nat. Prod. Res..

[48] Del Chierico F., Vernocchi P., Petrucca A., Paci P., Fuentes S., Praticò G., Capuani G., Masotti A., Reddel S., Russo A. (2015). Phylogenetic and Metabolic Tracking of Gut Microbiota during Perinatal Development. PLoS ONE.

[49] Dessì A., Briana D., Corbu S., Gavrili S., Cesare Marincola F., Georgantzi S., Pintus R., Fanos V., Malamitsi-Puchner A. (2018). Metabolomics of Breast Milk: The Importance of Phenotypes. Metabolites.

[50] Qannari E.M., Wakeling I., Courcoux P., MacFie H.J.H. (2000). Defining the Underlying Sensory Dimensions. Food Qual. Prefer..

