# Supplementary material for: Gut Microbiota Functional Traits, Blood pH, and Anti-GAD Antibodies Concur in the Clinical Characterization of T1D at Onset
Source: Int J Mol Sci. 2022 Sep 6;23(18):10256. doi: 10.3390/ijms231810256 (PMC9499637; doi:10.3390/ijms231810256)
Supplement: Supplementary file 1 [file ijms-23-10256-s001.zip › ijms-1871357-supplementary.pdf]

Supplementary materials

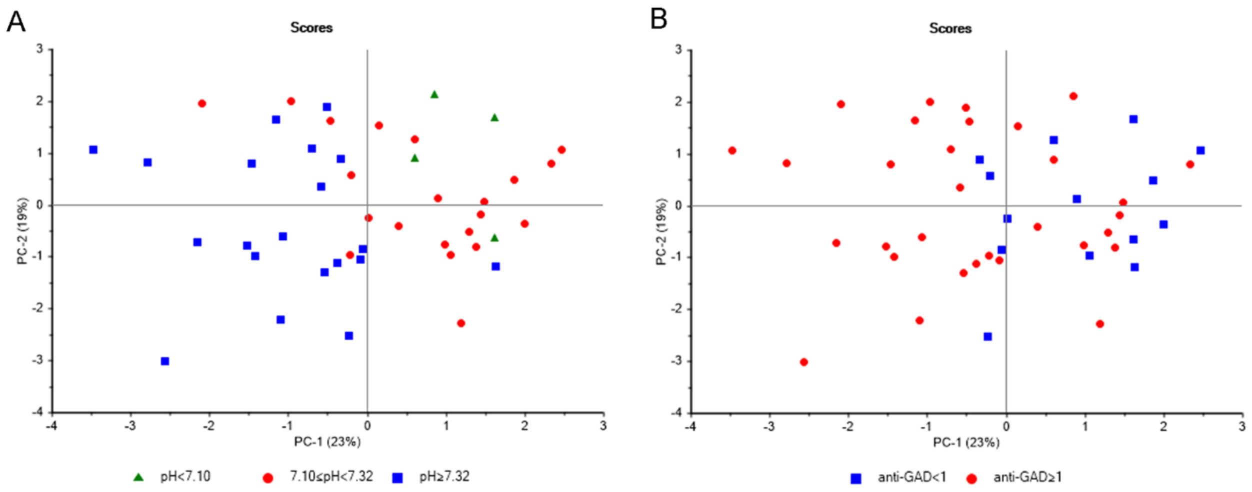

Figure S1. PCA score plots with labels based on severity thresholds identified for the clinical parameters blood pH and anti-GAD. Panel A: blue: blood pH≥7.32; red: 7.10≤blood pH<7.32; green: blood pH<7.10. Panel B: red anti-GAD≥1; blue: anti-GAD<1.

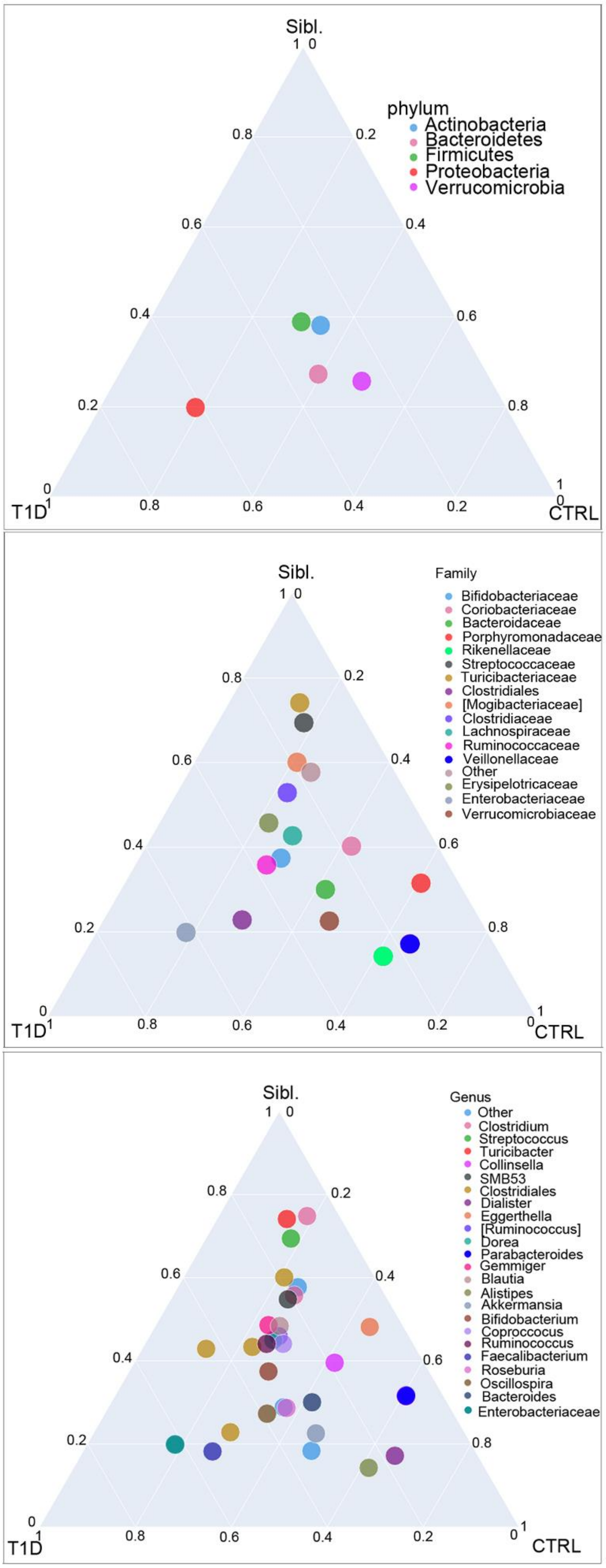

Figure S2. Ternary plot at phylum, family and genus levels. In figure are plotted the relative abundances of bacterial taxa shared amongst siblings, T1D patients and CTRL.

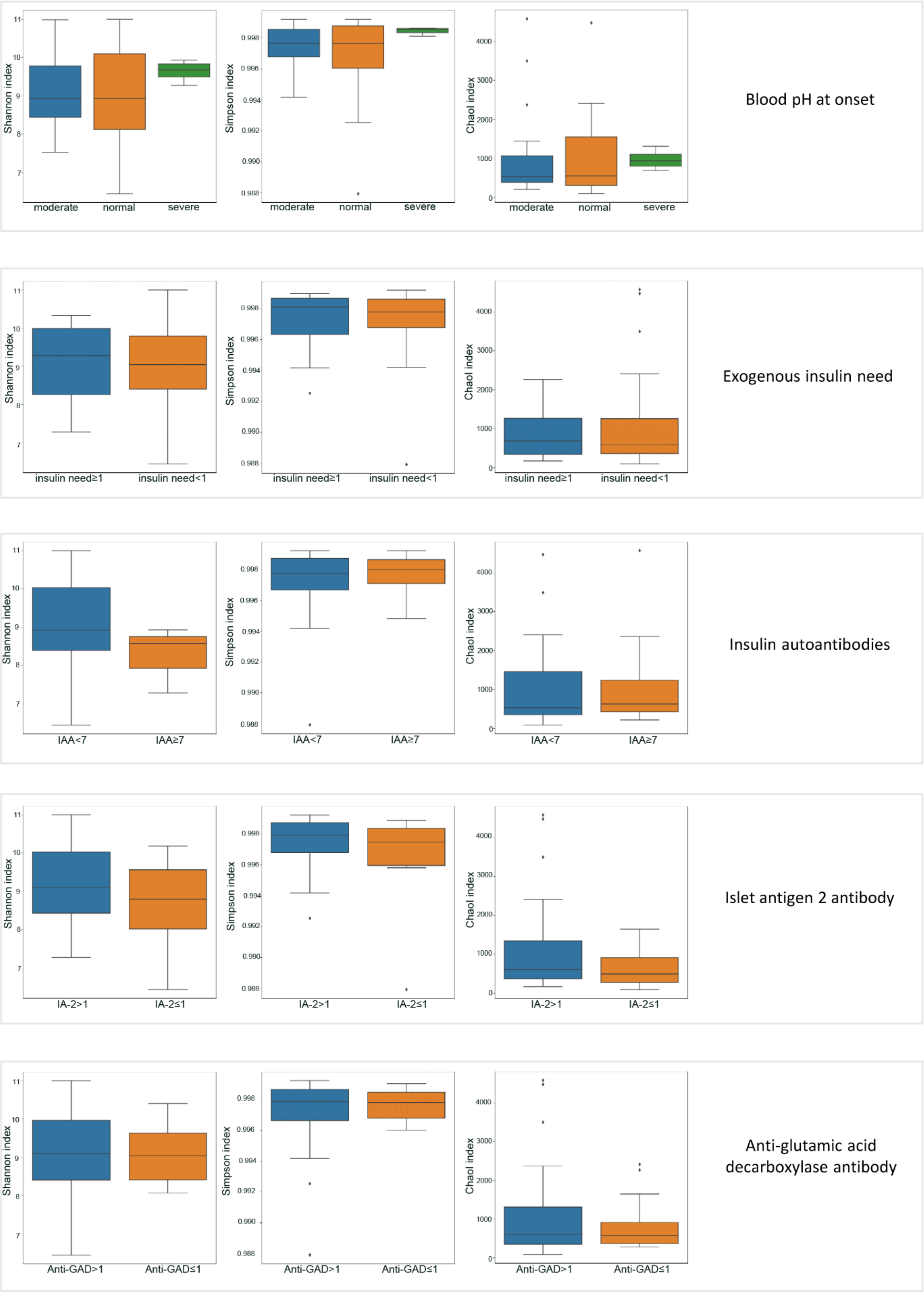

Figure S3. Alpha-diversity Shannon, Simpson and ChaoI indexes calculated grouping patients for each clinical parameter. Kruskal-Wallis test has been applied for each comparison.

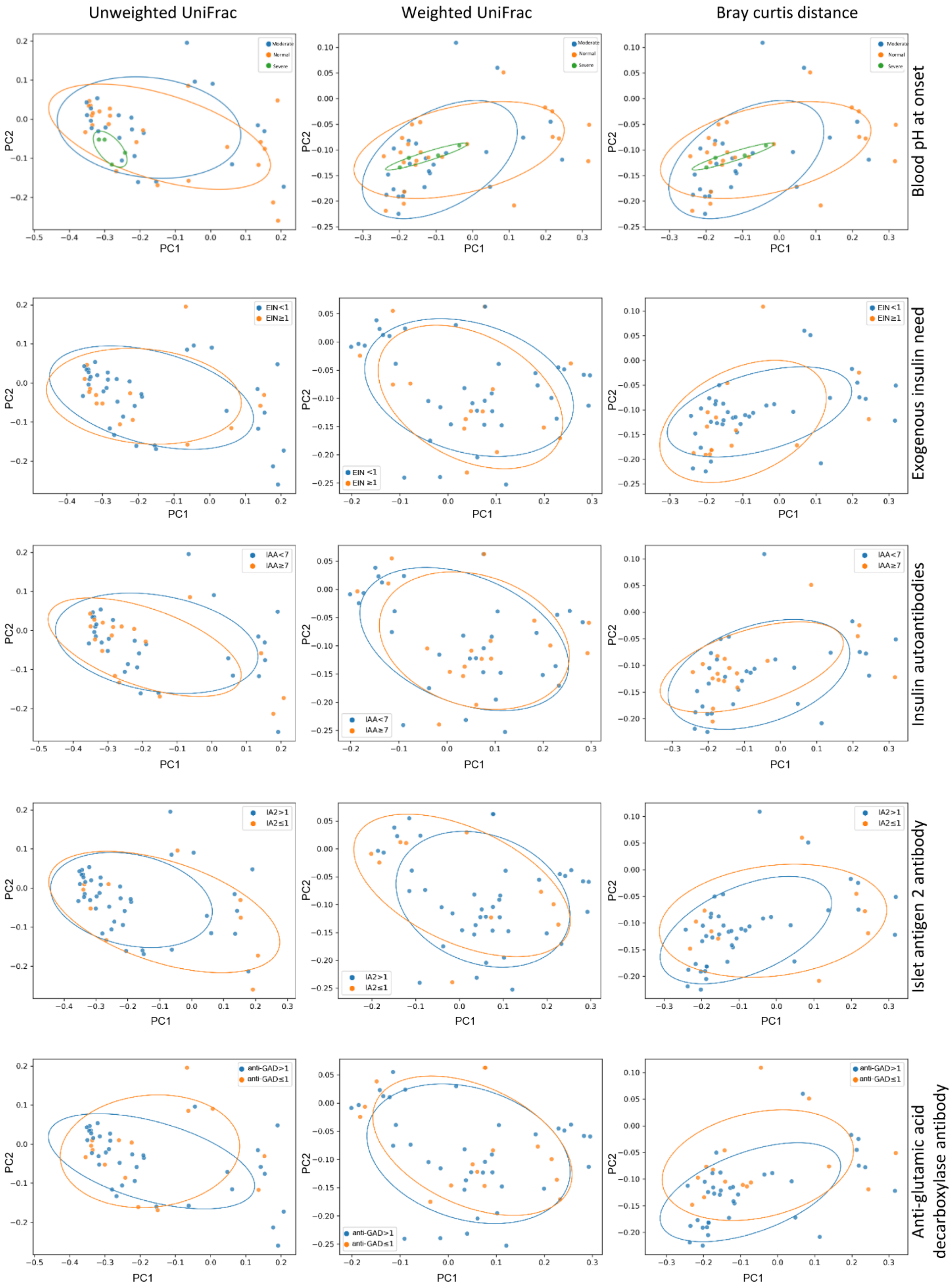

Figure S4. Beta-diversity PCoA plots on distance matrices calculated by Unweighted UniFrac, Weighted Unifrac and Bray Curtis algorithms grouping patients for each clinical parameter. Permanova analysis has been applied for each comparison.

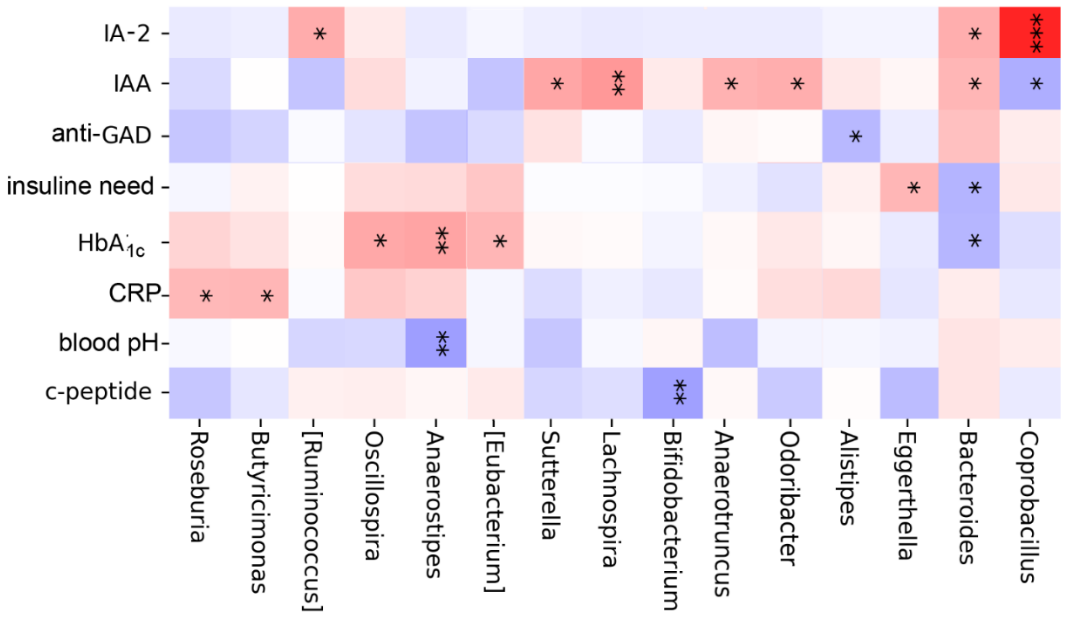

Figure S5. Microbiota and clinical features correlation. Pearson's correlation analysis of clinical feature values and relative abundance of microbial taxa. Blue and red shades indicate negative or positive correlations, respectively. The star symbol highlights statistically significant correlations (*p value <0.05; **p value<0.01; ***p value<0.0001). Reported correlations are selected on the bases of at least one correlation statistically significant (p value≤ 0.05).

Pearson's correlation analysis revealed a positive correlation between IA-2 antibodies and *Coprobacillus*, *Bacteroides*, and *Ruminococcus* (Lachnospiraceae). IAA antibodies positively correlated with *Sutterella*, *Lachnospira*, *Anaerotruncus*, *Odoribacter*, and *Bacteroides* and negatively with *Coprobacillus*. Anti-GAD antibodies negatively correlated with *Alistipes*. The insulin need correlated positively with *Eggerthella* and negatively with *Bacteroides*. HbA1c positively correlated with *Oscillospira*, *Anaerostipes*, *Eubacterium*, and negatively with *Bacteroides*. CRP was positively correlated with *Roseburia* and *Butyricimonas*. Blood pH levels were negatively correlated with *Anaerostipes*. C-peptide was negatively correlated with *Bifidobacterium*.

The positive correlation among *Bacteroides* and levels of IA-2 and IAA and negative with insulin need and HbA1c suggested a possible relationship of this genus with the early stage of disease, when antibody levels are increasing but the severity of diabetes, as measured by Hb1Ac and insulin need, is not yet advanced. Interestingly, the relative abundance of *Anaerostipes* increased to the decrease of blood pH levels, leading to presume a possible link of this microorganism with the ketoacidosis status of patients.

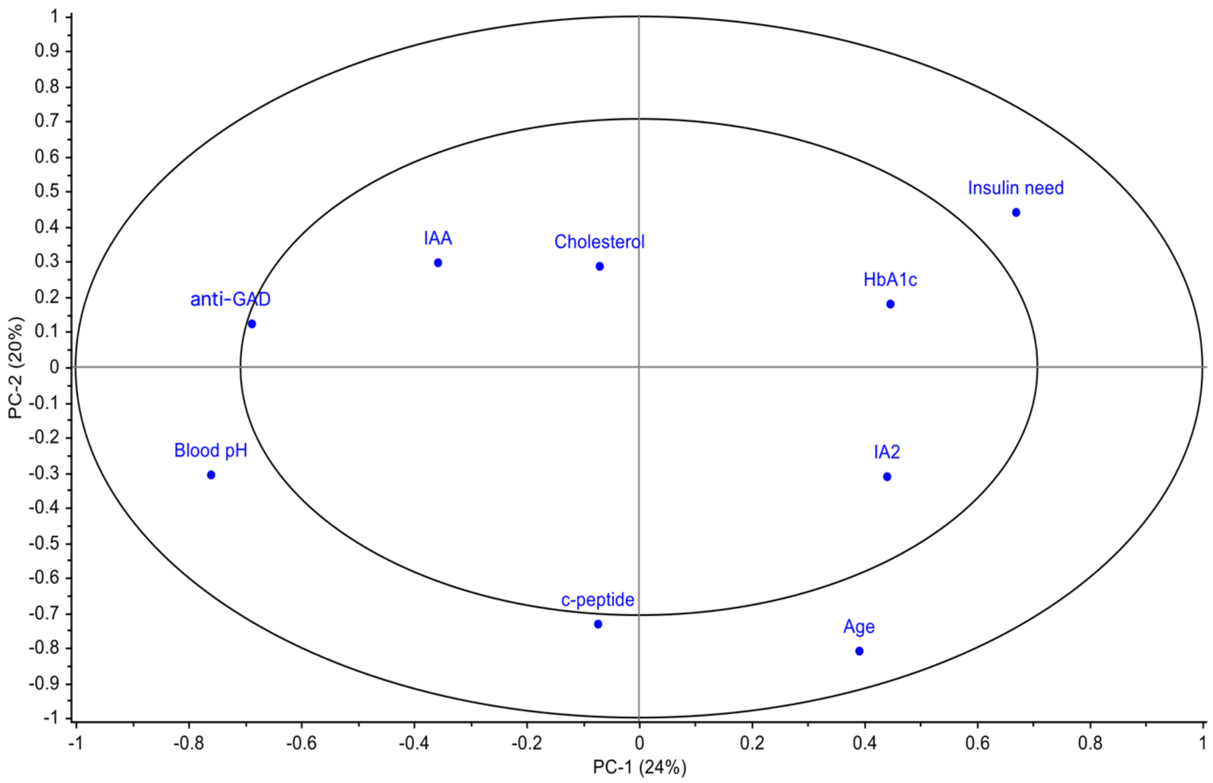

Figure S6. Principal component analysis (PCA) loadings plot performed on the clinical data matrix of T1D subset.

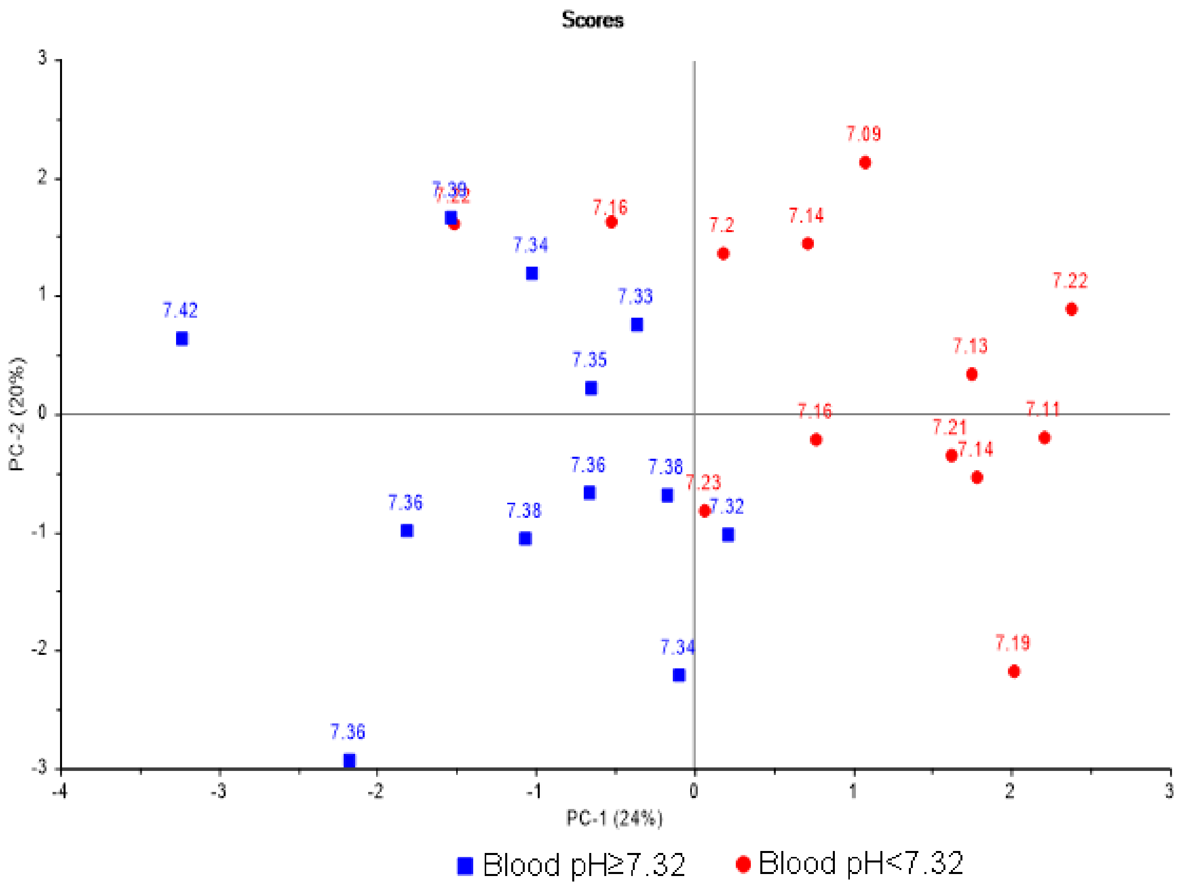

Figure S7. Principal component analysis (PCA) scores plot performed on clinical data for T1D subset. Red: T1D patients with blood pH<7.32; blue: T1D patients with blood pH≥7.32.

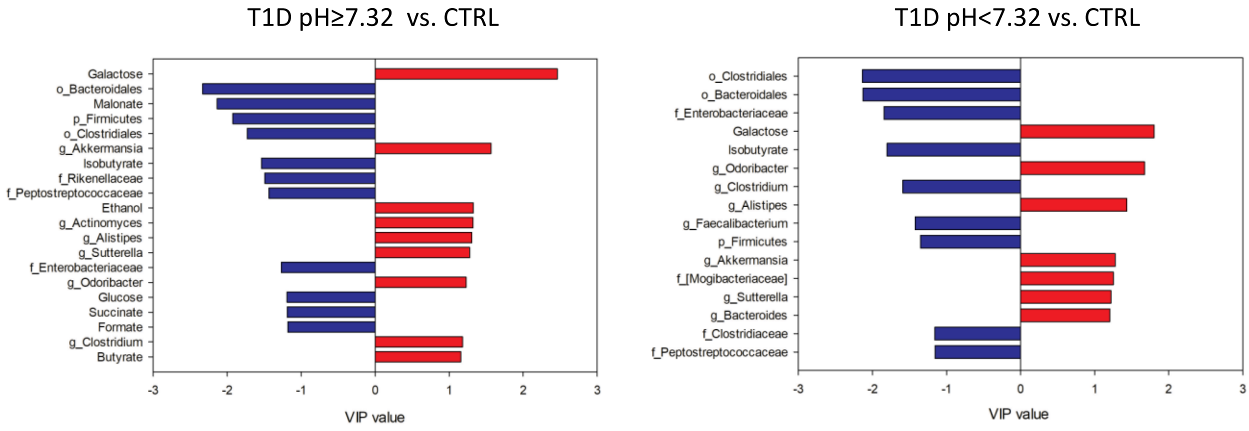

Figure S8. Variable Importance in Projection (VIP) values of the significant metabolites and ASVs in the omics-data integration analysis. In blue the feature's levels higher in T1D pH≥7.32 and T1D pH<7.32; in red the feature's levels higher in CTRL. In the comparison between T1D pH≥7.32 and CTRL, patients showed higher levels of malonate, isobutyrate, glucose, succinate, formate, Rikenellaceae, Peptostreptococcaceae, Enterobacteriaceae, Clostridiales, Bacteroidales and Firmicutes, while CTRL showed higher levels of galactose, ethanol, butyrate, *Akkermansia*, *Actinomyces*, *Alistipes*, *Sutterella*, *Odoribacter* and *Clostridium* (Clostridiaceae).

The features higher in T1D pH<7.32 in comparison with CTRL were isobutyrate, *Clostridium* (Lachnospiraceae), *Faecalibacterium*, Enterobacteriaceae, Clostridiaceae, Peptostreptococcaceae, Clostridiales, Bacteroidales and Firmicutes. CTRL showed higher levels of galactose, *Odoribacter*, *Alistipes*, *Akkermansia*, *Sutterella*, Mogibacteriaceae and *Bacteroides*.

Table S1 A. Kruskal-Wallis test amongst siblings, T1D patients and CTRL. For each group are reported the relative abundance average value of ASVs, and in bold the *p* values≤0.05 and FDR<0.1.

Phylum	Family	Genus	Sibling	T1D patients	CTRL	p value	FDR_P
Actinobacteria	Bifidobacteriaceae	*Bifidobacterium*	0.0424	0.0360	0.0358	0.126	0.233
Actinobacteria	Coriobacteriaceae	*Collinsella*	0.0106	0.0057	0.0121	**0.000**	**0.002**
Bacteroidetes	Bacteroidaceae	*Bacteroides*	0.1157	0.1445	0.1390	0.732	0.799
Bacteroidetes	Rikenellaceae	*Alistipes*	0.0068	0.0088	0.0228	0.571	0.653
Firmicutes	Enterococcaceae	*Enterococcus*	0.0413	0.0137	0.0028	0.864	0.902
Firmicutes	Streptococcaceae	*Streptococcus*	0.0432	0.0110	0.0168	**0.001**	**0.006**
Firmicutes	Clostridiales unk. family		0.0325	0.0322	0.0185	**0.000**	**0.000**
Firmicutes	Clostridiaceae	*Clostridium*	0.0112	0.0044	0.0055	**0.000**	**0.002**
Firmicutes	Lachnospiraceae		0.0146	0.0248	0.0156	**0.009**	**0.036**
Firmicutes	Lachnospiraceae	*Ruminococcus*	0.0165	0.0077	0.0109	0.050	0.121
Firmicutes	Lachnospiraceae	*Blautia*	0.0558	0.0272	0.0262	0.188	0.301
Firmicutes	Lachnospiraceae	*Coprococcus*	0.0227	0.0125	0.0156	0.042	0.111
Firmicutes	Lachnospiraceae	*Dorea*	0.0283	0.0235	0.0160	**0.015**	**0.046**
Firmicutes	Lachnospiraceae	*Roseburia*	0.0168	0.0130	0.0222	0.516	0.651
Firmicutes	Ruminococcaceae		0.0168	0.0243	0.0294	0.305	0.430
Firmicutes	Ruminococcaceae		0.0086	0.0168	0.0245	0.228	0.342
Firmicutes	Ruminococcaceae	*Faecalibacterium*	0.0240	0.0864	0.0368	0.569	0.653
Firmicutes	Ruminococcaceae	*Gemmiger*	0.0943	0.0519	0.0525	0.168	0.288
Firmicutes	Ruminococcaceae	*Oscillospira*	0.0160	0.0134	0.0223	0.392	0.522
Firmicutes	Ruminococcaceae	*Ruminococcus*	0.0403	0.0333	0.0292	0.062	0.135
Firmicutes	Veillonellaceae	*Dialister*	0.0090	0.0077	0.0424	**0.000**	**0.002**
Proteobacteria	Enterobacteriaceae		0.0525	0.1661	0.0485	0.085	0.171
Verrucomicrobia	Verrucomicrobiaceae	*Akkermansia*	0.1043	0.1039	0.1978	**0.014**	**0.046**

Table S1 B. Relative abundance values of *Akkermansia*, *Dialister*, *Dorea* and Clostridiales for each couple composed of the T1D patient and the relative sibling, compared to the CTRLs' mean value. In bold were highlighted the values lower than the mean of CTRL for *Akkermansia* and *Dialister*, higher than the mean of CTRL for *Dorea* and Clostridiales.

Akkermansia	**Sibling**	**T1D**	**Mean CTRL**
Couple 01	**0.007427**	**0.00639923**	0.1978
Couple 02	**0.00145528**	0.38080781	
Couple 03	**0.00598457**	0.24001653	
Couple 04	**0.17859146**	**0.00548357**	
Couple 05	0.58068875	**0.19049835**	
Couple 06	**0.00060077**	**0.00033585**	
Couple 07	**0.00055423**	**0**	
Couple 08	0.41345445	**0.00010904**	
Couple 09	**0.00074942**	**0.00186688**	
Couple 10	**0.00108764**	**0.027406**	
Couple 11	**0.00315965**	0.38168476	
Couple 12	**0.183344**	**0.138876**	
Couple 13	**0.04153661**	**0.04997096**	
Couple 14	**0.08073146**	**0.00491362**	

Dialister	**Sibling**	**T1D**	**Mean CTRL**
Couple 01	**0.00015**	**0**	0.0424
Couple 02	**0**	**0.00016499**	
Couple 03	**0.00248234**	**0.01046133**	
Couple 04	**0.00022949**	**0**	
Couple 05	**0.00103936**	**0**	
Couple 06	**0.00603563**	**0.00547236**	
Couple 07	**0.0018885**	**0.00077**	
Couple 08	**0.01054266**	**0.00021808**	
Couple 09	**0**	**0**	
Couple 10	**0.00111483**	**0.000359**	
Couple 11	**0.00070004**	**0.0045217**	
Couple 12	0.065164	**0.028426**	
Couple 13	0.04798856	0.05326983	
Couple 14	**0.00022769**	**0.00031701**	

Dorea	**Sibling**	**T1D**	**Mean CTRL**
Couple 01	**0.070056**	0.00853231	0.016
Couple 02	0.00336335	0.00062698	
Couple 03	**0.04229607**	**0.01797799**	
Couple 04	**0.05543373**	0.0028631	
Couple 05	0.00869595	0.00237309	
Couple 06	**0.02098498**	**0.01995338**	
Couple 07	**0.02580261**	0.00186082	
Couple 08	**0.05195505**	0.00043616	
Couple 09	0.00685445	**0.07776127**	
Couple 10	**0.0200397**	**0.044549**	
Couple 11	**0.04824611**	0.01320524	
Couple 12	0.008834	0.008386	
Couple 13	0.00551031	0.0059705	
Couple 14	0.01239505	0.01378982	

**Clostridiales**	**Sibling**	**T1D**	**Mean CTRL**
Couple 01	**0.028443**	**0.03997798**	0.0185
Couple 02	**0.04601541**	**0.0655029**	
Couple 03	**0.0290569**	**0.03347626**	
Couple 04	**0.06889694**	**0.03741447**	
Couple 05	0.01016838	**0.04569355**	
Couple 06	**0.04733496**	**0.05187878**	
Couple 07	**0.02962066**	**0.02499278**	
Couple 08	**0.03291445**	**0.05866318**	
Couple 09	**0.03697746**	0.0184783	
Couple 10	**0.04842701**	0.017086	
Couple 11	**0.03307224**	**0.03071315**	
Couple 12	**0.039772**	**0.026204**	
Couple 13	**0.02983591**	**0.07808108**	
Couple 14	0.0160666	**0.03479157**	

Table S2. Kruskal-Wallis test on clinical features. For each group are reported the relative abundance average value of ASVs and in bold the *p* values≤0.05 and FDR≤0.1.

			Blood pH at onset					Exogenous insulin need			
**Phylum**	**Family**	**Genus**	**severe**	**moderate**	**normal**	**p value**	**FDR**	**FI>=1**	**FI<1**	**p value**	**FDR**
Actinobacteria	Bifidobacteriaceae	*Bifidobacterium*	0.0257	0.0400	0.0364	0.837	0.862	0.0289	0.0405	0.965	0.982
Actinobacteria	Coriobacteriaceae	*Collinsella*	0.0016	0.0044	0.0083	0.401	0.752	0.0032	0.0072	0.334	0.877
Bacteroidetes	Bacteroidaceae	*Bacteroides*	0.1590	0.1161	0.1638	0.467	0.752	0.1241	0.1492	0.982	0.982
Bacteroidetes	Rikenellaceae	*Alistipes*	0.0094	0.0077	0.0086	0.125	0.599	0.0076	0.0085	0.947	0.982
Firmicutes	Enterococcaceae	*Enterococcus*	0.0000	0.0008	0.0302	0.470	0.752	0.0003	0.0202	0.722	0.964
Firmicutes	Streptococcaceae	*Streptococcus*	0.0125	0.0053	0.0173	0.467	0.752	0.0098	0.0122	0.842	0.982
Firmicutes	Clostridiaceae	*Clostridium*	0.0016	0.0050	0.0048	0.614	0.862	0.0056	0.0042	0.413	0.877
Firmicutes	Lachnospiraceae	*Blautia*	0.0196	0.0202	0.0372	0.246	0.752	0.0282	0.0281	0.426	0.877
Firmicutes	Lachnospiraceae	*Coprococcus*	0.0148	0.0089	0.0160	0.862	0.862	0.0085	0.0144	0.595	0.953
Firmicutes	Lachnospiraceae	*Dorea*	0.0451	0.0221	0.0225	0.851	0.862	0.0289	0.0223	0.791	0.982
Firmicutes	Lachnospiraceae	*Roseburia*	0.0044	0.0108	0.0136	0.631	0.862	0.0088	0.0127	0.095	0.877
Firmicutes	Lachnospiraceae	*Ruminococcus*	0.0153	0.0085	0.0064	0.418	0.752	0.0077	0.0082	0.595	0.953
Firmicutes	Lachnospiraceae		0.0342	0.0263	0.0231	0.230	0.752	0.0262	0.0251	0.278	0.877
Firmicutes	Ruminococcaceae	*Faecalibacterium*	0.2615	0.0870	0.0632	0.039	0.475	0.0400	0.1101	0.259	0.877
Firmicutes	Ruminococcaceae	*Gemmiger*	0.0153	0.0373	0.0718	0.419	0.752	0.0522	0.0515	0.543	0.953
Firmicutes	Ruminococcaceae	*Oscillospira*	0.0095	0.0174	0.0106	0.722	0.862	0.0188	0.0114	0.215	0.877
Firmicutes	Ruminococcaceae	*Ruminococcus*	0.0168	0.0315	0.0385	0.833	0.862	0.0481	0.0278	0.439	0.877
Firmicutes	Ruminococcaceae		0.0594	0.0178	0.0253	0.059	0.475	0.0262	0.0241	0.642	0.963
Firmicutes	Clostridiales		0.0369	0.0367	0.0280	0.459	0.752	0.0343	0.0320	0.723	0.964
Firmicutes	Veillonellaceae	*Dialister*	0.0023	0.0055	0.0100	0.725	0.862	0.0041	0.0086	0.343	0.877
Proteobacteria	Enterobacteriaceae		0.0699	0.2562	0.1180	0.058	0.475	0.2373	0.1517	0.199	0.877
Verrucomicrobia	Verrucomicrobiaceae	*Akkermansia*	0.0953	0.0779	0.0939	0.305	0.752	0.1037	0.0801	0.192	0.877

			insulin autoantibodies				islet antigen 2 antibody			
**Phylum**	**Family**	**Genus**	**IAA<7**	**IAA≥7**	**p value**	**FDR**	**IA-2≤1**	**IA-2>1**	**p value**	**FDR**
Actinobacteria	Bifidobacteriaceae	*Bifidobacterium*	0.0278	0.0533	0.340	0.975	0.0655	0.0308	0.394	0.847
Actinobacteria	Coriobacteriaceae	*Collinsella*	0.0077	0.0031	0.933	0.975	0.0075	0.0057	0.346	0.847
Bacteroidetes	Bacteroidaceae	*Bacteroides*	0.1210	0.1782	0.263	0.975	0.1251	0.1458	0.757	0.847
Bacteroidetes	Rikenellaceae	*Alistipes*	0.0090	0.0071	0.934	0.975	0.0139	0.0070	0.624	0.847
Firmicutes	Enterococcaceae	*Enterococcus*	0.0228	0.0001	0.124	0.975	0.0638	0.0034	0.202	0.847
Firmicutes	Streptococcaceae	*Streptococcus*	0.0105	0.0132	0.901	0.975	0.0121	0.0114	0.408	0.847
Firmicutes	Clostridiaceae	*Clostridium*	0.0049	0.0043	0.443	0.975	0.0029	0.0050	0.679	0.847
Firmicutes	Lachnospiraceae	*Blautia*	0.0315	0.0224	0.917	0.975	0.0505	0.0231	0.535	0.847

Firmicutes	Lachnospiraceae	*Coprococcus*	0.0159	0.0072	0.245	0.975	0.0108	0.0132	0.776	0.847
Firmicutes	Lachnospiraceae	*Dorea*	0.0215	0.0289	0.803	0.975	0.0235	0.0243	0.757	0.847
Firmicutes	Lachnospiraceae	*Roseburia*	0.0141	0.0071	0.548	0.975	0.0050	0.0130	0.519	0.847
Firmicutes	Lachnospiraceae	*Ruminococcus*	0.0090	0.0064	0.507	0.975	0.0085	0.0079	0.394	0.847
Firmicutes	Lachnospiraceae		0.0217	0.0318	0.494	0.975	0.0467	0.0206	0.016	0.392
Firmicutes	Ruminococcaceae	*Faecalibacterium*	0.0750	0.1160	0.619	0.975	0.0181	0.1062	0.642	0.847
Firmicutes	Ruminococcaceae	*Gemmiger*	0.0572	0.0421	0.604	0.975	0.0234	0.0580	0.439	0.847
Firmicutes	Ruminococcaceae	*Oscillospira*	0.0128	0.0149	0.351	0.975	0.0109	0.0142	0.661	0.847
Firmicutes	Ruminococcaceae	*Ruminococcus*	0.0374	0.0270	0.820	0.975	0.0107	0.0387	0.134	0.847
Firmicutes	Ruminococcaceae		0.0190	0.0346	0.158	0.975	0.0326	0.0229	0.857	0.894
Firmicutes	Clostridiales		0.0298	0.0376	0.534	0.975	0.0375	0.0315	0.245	0.847
Firmicutes	Veillonellaceae	*Dialister*	0.0081	0.0059	0.975	0.975	0.0144	0.0057	0.706	0.847
Proteobacteria	Enterobacteriaceae		0.2043	0.1277	0.828	0.975	0.1358	0.1852	0.561	0.847
Verrucomicrobia	Verrucomicrobiaceae	*Akkermansia*	0.0916	0.0785	0.967	0.975	0.0953	0.0849	0.959	0.959
			**anti-glutamic acid decarboxylase antibody**				**Blood pH at onset**			
**Phylum**	**Family**	**Genus**	**anti-GAD≤1**	**anti-GAD>1**	**p value**	**FDR**	**pH<7.32**	**pH≥7.32**	**p value**	**FDR**
Actinobacteria	Bifidobacteriaceae	*Bifidobacterium*	0.0416	0.0352	0.588	0.796	0.0312	0.0201	0.057	0.344
Actinobacteria	Coriobacteriaceae	*Collinsella*	0.0090	0.0047	0.597	0.796	0.0010	0.0007	0.099	0.396
Bacteroidetes	Bacteroidaceae	*Bacteroides*	0.1274	0.1485	0.879	0.953	0.0993	0.2781	0.181	0.543
Bacteroidetes	Rikenellaceae	*Alistipes*	0.0180	0.0040	**0.010**	**0.078**	0.0030	0.0051	0.778	0.778
Firmicutes	Enterococcaceae	*Enterococcus*	0.0041	0.0191	0.576	0.796	0.0004	0.0019	0.346	0.597
Firmicutes	Streptococcaceae	*Streptococcus*	0.0071	0.0135	0.447	0.759	0.0044	0.0020	0.307	0.597
Firmicutes	Clostridiaceae	*Clostridium*	0.0015	0.0060	0.113	0.579	0.0077	0.0010	0.020	0.344
Firmicutes	Lachnospiraceae	*Blautia*	0.0351	0.0251	0.318	0.695	0.0102	0.0086	0.778	0.778
Firmicutes	Lachnospiraceae	*Coprococcus*	0.0178	0.0105	0.241	0.644	0.0065	0.0040	0.439	0.619
Firmicutes	Lachnospiraceae	*Dorea*	0.0215	0.0254	0.398	0.759	0.0282	0.0063	0.398	0.597
Firmicutes	Lachnospiraceae	*Roseburia*	0.0210	0.0074	**0.009**	**0.078**	0.0055	0.0069	0.526	0.702
Firmicutes	Lachnospiraceae	*Ruminococcus*	0.0070	0.0085	0.914	0.953	0.0120	0.0033	0.049	0.344
Firmicutes	Lachnospiraceae		0.0259	0.0252	0.448	0.759	0.0307	0.0167	0.078	0.376
Firmicutes	Ruminococcaceae	*Faecalibacterium*	0.2109	0.0367	**0.004**	**0.078**	0.0789	0.1181	0.324	0.597
Firmicutes	Ruminococcaceae	*Gemmiger*	0.0346	0.0592	0.879	0.953	0.0416	0.0206	0.725	0.778
Firmicutes	Ruminococcaceae	*Oscillospira*	0.0186	0.0113	0.288	0.691	0.0142	0.0106	0.673	0.778
Firmicutes	Ruminococcaceae	*Ruminococcus*	0.0590	0.0224	0.165	0.579	0.0288	0.0120	0.260	0.597
Firmicutes	Ruminococcaceae		0.0230	0.0254	0.474	0.759	0.0294	0.0204	0.398	0.597
Firmicutes	Clostridiales		0.0277	0.0348	0.633	0.800	0.0508	0.0246	0.049	0.344
Firmicutes	Veillonellaceae	*Dialister*	0.0156	0.0037	0.186	0.579	0.0067	0.0147	0.168	0.543

Proteobacteria	Enterobacteriaceae		0.0949	0.2120	0.193	0.579	0.2461	0.2006	0.360	0.597
Verrucomicrobia	Verrucomicrobiaceae	*Akkermansia*	0.0380	0.1083	0.140	0.579	0.1121	0.1096	0.751	0.778
			**Blood pH at onset**				**Blood pH at onset**			
**Phylum**	**Family**	**Genus**	**pH<7.32**	**CTRL**	**p value**	**FDR**	**pH≥7.32**	**CTRL**	**p value**	**FDR**
Actinobacteria	Bifidobacteriaceae	*Bifidobacterium*	0.0312	0.0374	0.673	0.897	0.0201	0.0374	0.070	0.186
Actinobacteria	Coriobacteriaceae	*Collinsella*	0.0010	0.0117	0.024	0.139	0.0007	0.0117	0.008	0.137
Bacteroidetes	Bacteroidaceae	*Bacteroides*	0.0993	0.1123	0.324	0.519	0.2781	0.1123	0.070	0.186
Bacteroidetes	Rikenellaceae	*Alistipes*	0.0030	0.0145	0.833	0.908	0.0051	0.0145	1.000	1.000
Firmicutes	Enterococcaceae	*Enterococcus*	0.0004	0.0000	0.016	0.124	0.0019	0.0000	0.061	0.186
Firmicutes	Streptococcaceae	*Streptococcus*	0.0044	0.0501	0.778	0.908	0.0020	0.0501	0.289	0.497
Firmicutes	Clostridiaceae	*Clostridium*	0.0077	0.0085	0.673	0.897	0.0010	0.0085	0.023	0.137
Firmicutes	Lachnospiraceae	*Blautia*	0.0102	0.0153	0.324	0.519	0.0086	0.0153	0.290	0.497
Firmicutes	Lachnospiraceae	*Coprococcus*	0.0065	0.0132	0.181	0.434	0.0040	0.0132	0.028	0.137
Firmicutes	Lachnospiraceae	*Dorea*	0.0282	0.0260	0.944	0.985	0.0063	0.0260	0.406	0.649
Firmicutes	Lachnospiraceae	*Roseburia*	0.0055	0.0098	0.231	0.463	0.0069	0.0098	0.496	0.709
Firmicutes	Lachnospiraceae	*Ruminococcus*	0.0120	0.0114	0.526	0.789	0.0033	0.0114	0.013	0.137
Firmicutes	Lachnospiraceae		0.0307	0.0210	0.049	0.167	0.0167	0.0210	0.650	0.709
Firmicutes	Ruminococcaceae	*Faecalibacterium*	0.0789	0.0220	0.260	0.480	0.1181	0.0220	0.705	0.736
Firmicutes	Ruminococcaceae	*Gemmiger*	0.0416	0.0340	0.832	0.908	0.0206	0.0340	0.650	0.709
Firmicutes	Ruminococcaceae	*Oscillospira*	0.0142	0.0150	0.725	0.908	0.0106	0.0150	0.650	0.709
Firmicutes	Ruminococcaceae	*Ruminococcus*	0.0288	0.0131	1.000	1.000	0.0120	0.0131	0.226	0.453
Firmicutes	Ruminococcaceae		0.0294	0.0401	0.067	0.201	0.0204	0.0401	0.597	0.709
Firmicutes	Clostridiales		0.0508	0.0149	0.005	0.109	0.0246	0.0149	0.112	0.270
Firmicutes	Veillonellaceae	*Dialister*	0.0067	0.0264	0.009	0.109	0.0147	0.0264	0.034	0.137
Proteobacteria	Enterobacteriaceae		0.2461	0.0126	0.029	0.139	0.2006	0.0126	0.597	0.709
Verrucomicrobia	Verrucomicrobiaceae	*Akkermansia*	0.1121	0.3042	0.049	0.167	0.1096	0.3042	0.034	0.137

Table S3. List of metabolites identified in stools by means of NMR spectroscopy. The univocal assignment was performed on the basis of 2D-experiments: COSY, TOCSY, HSQC and HMBC.

Nr.	Metabolite	Group	^{1}H (ppm)	Molteplicity
1.	2-methylbutyrate	CH_3	0.86	
			1.05	t
			1.40	d
			1.49	m
			2.20	
2.	Valerate	CH_3	0.89	
			1.31	t
			1.54	m
			2.18	
3.	Isovalerate	2 CH_3	0.91	d
		CH	1.95	
		CH_2	2.06	d
4.	Ile		0.94	t
			1.01	d
		CH_3	1.28	m
		CH_3	1.48	
			1.97	m
			3.67	d
5.	Leu	CH_3	0.96	d
		CH_3	0.97	d
		CH	1.72	m
		CH_2	1.72	m
		α-CH	3.74	m
6.	Val	CH_3	0.99	d
		CH_3	1.05	d
		CH	2.27	m
		α-CH	3.61	d
7.	Isobutyrate	CH_3, CH_3’	1.07	d
		CH	2.40	m
8.	2-oxoisovalerate	2 CH_3	1.13	d
		CH	3.04	m
9.	Ethanol	CH_3	1.18	t
		CH_2	3.65	m
10.	Thr	CH_3	1.33	d
		α-CH	3.59	d
		CH	4.27	m
11.	Lactate	CH_3	1.33	d
		CH	4.13	m
12.	Ala	CH_3	1.48	d
		α-CH	3.79	q
13.	2-aminoisobutyrate	CH_3, CH_3’	1.47	s
14.	Butyrate	CH_3	0.90	t
		β-CH_2	1.56	m
		α-CH_2	2.16	t
15.	Lys	ϒ-CH_2	1.46	
		δ-CH_2	1.72	m
			1.94	
		ε-CH_2	3.04	
		α-CH	3.76	
16.	Acetate	CH_3	1.92	s
17.	Propionate	CH_3	1.06	t

		CH_2	2.18	m
18.	Glu	β-CH	2.06	
		β-CH’	2.12	
		ϒ-CH_2	2.36	pt
		α-CH	3.76	
19.	Succinate	α,β-CH_2	2.41	s
20.	Gln	β-CH_2	2.13	m
		ϒ-CH_2	2.46	m
		α-CH	3.78	
21.	Asp	β-CH	2.68	dd
		β-CH’	2.81	dd
		α-CH	3.91	dd
22.	DMA	CH_3,CH_3’	2.75	s
23.	TMA	3 CH_3	2.91	s
24.	Malonate	CH_2	3.12	s
25.	Gly	CH_2	3.56	s
26.	Trigonelline	CH_3	4.42	s
27.	α-Glucose		3.41	
			3.55	
			3.70	
			3.83	
		CH	5.23	d
28.	α-Galactose		3.83	
			4.00	
		CH	5.27	d
29.	Uracil	CH	5.80	d
		CH	7.55	d
30.	Orotate	CH	6.19	s
31.	Fumarate	CH, CH’	6.52	s
32.	4-hydroxyphenilacetate	CH-2, CH-6	6.87	pd
		CH-3, CH-5	7.19	pd
33.	Tyr	CH-1, CH-5	6.90	d
		CH-2, CH-4	7.20	d
34.	Phe	5 CH ring	7.38	m
35.	Hypoxanthine	CH	8.19	s
		CH	8.21	s
36.	Guanine	CH	7.90	s
37.	Formate	CH	8.46	s

s: singlet; d: doublet; t: triplet; m: multiplet; pd: pseudo doublet, pt: pseudo triplet.

Table S4. Classification analyses on the: metabolomic, metagenomic and omics data integration datasets (low level fusion analysis performed on the two matrices jointly elaborated).

Class	LVopt[1]	Accuracy (%)	Mean Class. Err. (%)	Sensitivity (%)	Specificity (%)
**Metabolomic dataset**					
T1D	8±2	66.9±5.7	34.8±6.1	75.5±5.9	54.9±10.4
CTRL				54.9±10.4	75.5±5.9
T1DpH<7.32	7±2	61.0±6.8	39.8±6.9	56.5±8.6	64.0±7.9
CTRL				64.0±7.9	56.5±8.6
T1DpH≥7.32	2±3	58.6±4.9	40.9±5.1	62.5±7.2	55.8±5.5
CTRL				55.8±5.5	62.5±7.2
T1DpH≥7.32	3±2	55.5±5.6	44.5±5.6	56.2±8.9	54.9±6.4
T1DpH<7.32				54.9±6.4	56.2±8.9
**Metagenomic dataset**					
**Class**	**LVopt**	**Accuracy (%)**	**Mean Class. Err. (%)**	**Sensitivity (%)**	**Specificity (%)**
T1D	8±2	72.8±4.1	27.4±4.1	74.0±5.1	71.2±5.5
CTRL				71.2±5.5	74.0±5.1
T1DpH<7.32	6±2	68.2±4.6	32.3±4.6	65.2±7.3	70.2±6.2
CTRL				70.2±6.2	65.2±7.3
T1DpH≥7.32	8±2	78.3±3.9	23.1±4.3	68.0±7.5	85.8±3.2
CTRL				85.8±3.2	68.0±7.5
T1DpH≥7.32	3±3	49.4±6.3	50.3±6.4	57.2±7.1	42.3±9.4
T1DpH<7.32				42.3±9.4	57.2±7.1
**Omics Data Integration**					
**Class**	**LVopt**	**Accuracy (%)**	**Mean Class. Err. (%)**	**Sensitivity (%)**	**Specificity (%)**
T1D	4±1	91.3±3.2	9.2±3.4	93.8±3.1	87.8±5.8
CTRL				87.8±5.8	93.8±3.1
T1DpH<7.32	3±2	90.2±2.5	11.6±2.7	79.2±4.5	97.6±2.8
CTRL				97.6±2.8	79.2±4.5
T1DpH≥7.32	4±2	91.7±2.4	8.8 ±2.5	87.8±3.8	94.4±3.4
CTRL				94.4±3.4	87.8±3.8
T1DpH≥7.32	1±1	67.8±3.6	32.5±3.7	60.0±6.5	75.1±3.3
T1DpH<7.32				75.1±3.3	60.0±6.5

[1]optimal latent variable

Table S5. Comparisons between: T1D vs. CTRL, T1DpH≥7.32 vs. CTRL, T1DpH<7.32 vs. CTRL and T1DpH≥7.32 vs. T1DpH<7.32. Data were expressed by median, 25th percentile and 75th percentile. Significant metabolites and ASVs for the multi-block analysis were analyzed and reported below. Mann Whitney's u-test was performed. Bonferroni-corrected *p* values lower than 0.05 (*) were considered significant.

	T1D			CTRL			
	**Median**	**25%**	**75%**	**Median**	**25%**	**75%**	**p value**
Isobutyrate	0.701	0.571	1.145	0.604	0.153	0.863	0.044*
Ethanol	0.197	0.0839	0.344	0.329	0.238	0.584	0.024*
Butyrate	4.164	1.528	5.592	6.061	4.537	10.215	0.009*
Malonate	0.784	0.429	1.22	0.466	0.297	0.986	0.228
Succinate	0.246	0.175	0.474	0.389	0.228	0.701	0.058
Galactose	0.121	0.0948	0.188	0.334	0.236	0.465	<0.001*
Bacteroidales	0.242	0.0812	0.568	0	0	0	<0.001*
Alistipes	0.246	0.0344	0.713	0.722	0.0259	5.74	0.209
Odoribacter	0.00542	0	0.0505	0.00691	0	0.284	0.222
Clostridiales	3.07	1.55	5.67	0.679	0.181	1.98	<0.001*
Sutterella	0.0164	0	0.0318	0.032	0	0.153	0.286
Enterobacteriaceae	2.45	0.13	44.2	0.634	0.0353	3.37	0.03*
Akkermansia	0.971	0.0445	21.5	26.1	2.93	50.5	0.011*
Lachnospiraceae; Clostridium'	0.159	0.0405	0.699	0.00256	0	0.18	0.002*
Actinomyces	0	0	0.0178	0.00208	0	0.128	0.199
[Mogibacteriaceae]	0.124	0.033	0.324	0.18	0.0689	1.138	0.209
Adlercreutzia	0.00418	0	0.0127	0.027	0	0.152	0.033*
Collinsella	0.0244	0	0.11	0.2	0.0415	0.987	0.016*
Turicibacter	0.00956	0	0.0491	0.0132	0	0.246	0.425
	**T1DpH≥7.32**			**CTRL**			
	**Median**	**25%**	**75%**	**Median**	**25%**	**75%**	**p value**
Ethanol	0.212	0.0846	0.331	0.381	0.273	0.612	0.023*
Butyrate	4.298	1.643	5.82	6.716	5.121	14.728	0.034*
Galactose	0.138	0.096	0.199	0.368	0.294	0.555	<0.001*
Isobutyrate	1.023	0.422	1.488	0.402	0.134	0.914	0.057
Malonate	1.22	0.729	1.745	0.632	0.314	0.986	0.019*
Succinate	0.315	0.181	0.502	0.417	0.288	1.049	0.257
Glucose	0.324	0.162	0.57	1.23	1.008	2.426	0.023*
Formate	0.119	0.0889	0.187	0.0658	0.0375	0.128	0.07
Bacteroidales	0.444	0.139	0.799	0	0	0	<0.001*
Alistipes	0.324	0.0151	0.929	0.0759	0.0154	2.29	1
Odoribacter	0.032	0	0.0785	0	0	0.163	0.686
Firmicutes	0.0315	0.0047	0.0891	0	0	0	<0.001*
Clostridiales	1.94	1.09	3.89	1.03	0.227	2.74	0.112
Sutterella	0.0128	0	0.0441	0.00222	0	0.105	0.937
Enterobacteriaceae	0.909	0.0911	36.7	1.09	0.0477	2.05	0.597
Akkermansia	0.547	0.0179	26.1	28.8	12.6	47.6	0.039*
Rikenellaceae	0.263	0.0157	0.578	0.0218	0	0.155	0.08
Peptostreptococcaceae	0.0199	0.00388	0.0618	0	0	0	0.004*
Actinomyces	0.00314	0	0.0117	0.0589	0	0.171	0.079
Clostridiaceae; *Clostridium*	0.0854	0.0327	0.151	0.318	0.117	1.91	0.023*
	**T1DpH<7.32**			**CTRL**			
	**Median**	**25%**	**75%**	**Median**	**25%**	**75%**	**p value**
Ethanol	0.143	0.054	0.318	0.308	0.232	0.601	0.123
Isobutyrate	0.876	0.667	1.026	0.628	0.159	0.89	0.029*
Galactose	0.12	0.0594	0.177	0.313	0.175	0.41	0.007*
Malonate	0.549	0.166	0.606	0.454	0.296	0.966	0.445
Butyrate	4.273	2.453	5.785	6.662	4.077	11.101	0.158
Bacteroidales	0.155	0.0438	0.237	0	0	0	<0.001*

Alistipes	0.246	0.0361	0.604	0.0922	0.0158	1.05	0.622
Odoribacter	0	0	0.0231	0	0	0.104	0.693
Firmicutes	0.0651	0.0397	0.335	0	0	0	<0.001*
Clostridiales	4.57	2.96	6.55	1.18	0.265	2.57	0.002*
Sutterella	0.0141	0	0.06	0.00443	0	0.0843	0.89
Enterobacteriaceae	8.2	0.558	56	1.02	0.0515	1.66	0.023*
Akkermansia	5.73	0.0542	19.05	26.788	0.363	62.394	0.048*
Lachnospiraceae; *Clostridium*	0.577	0.13	1.897	0.031	0	0.276	0.011*
Faecalibacterium	0.818	0.141	2.699	1.546	0.572	2.874	0.25
Clostridiaceae	0.0545	0.0231	0.267	0.0183	0	0.318	0.188
_Peptostreptococcaceae	0.0379	0.0152	0.164	0	0	0	<0.001*
[Mogibacteriaceae]	0.166	0.0132	0.745	0.759	0.151	2.104	0.061
Bacteroides	9.021	1.527	16.072	2.94	0.215	14.881	0.45
	**T1DpH≥7.32**			**T1DpH<7.32**			
	**Median**	**25%**	**75%**	**Median**	**25%**	**75%**	**p value**
DMA	0.0548	0.0407	0.0625	0.0578	0.0447	0.0947	0.514
Uracil	1.061	0.777	1.1	0.767	0.528	0.854	0.017*
Guanine	0.534	0.504	0.586	0.363	0.233	0.429	0.009*
Formate	0.119	0.0906	0.163	0.0491	0.0286	0.0871	0.002*
2-methylbutyrate	1.323	0.967	1.569	0.933	0.752	1.298	0.074
Isovalerate	1.425	1.107	1.552	1.09	0.877	1.443	0.151
2-Aminoisobutyrate	0.895	0.561	1.353	0.584	0.493	0.901	0.082
Propionate	9.649	6.693	13	7.01	2.721	9.116	0.113
Glu	4.942	4.147	5.607	3.624	2.604	5.352	0.078
Malonate	1.114	0.793	1.564	0.583	0.21	0.801	0.015*
Fumarate	0.0618	0.0411	0.102	0.0493	0.0244	0.0568	0.059
Hypoxanthine	0.391	0.23	0.511	0.272	0.204	0.338	0.042*
Coriobacteriaceae	0.0408	0.0193	0.123	0.0233	0.0142	0.0833	0.252
Eggerthella	0	0	0.00949	0.0496	0.00886	0.222	0.004*
Bacteroidales	0.444	0.198	0.8	0.192	0.0519	0.248	0.064
Bacteroides	14	3.01	43	9.02	1.36	14.9	0.192
Parabacteroides	0.292	0.035	0.884	0.0782	0.0208	0.352	0.211
Rikenellaceae	0.263	0.0208	0.624	0.0428	0.00661	0.185	0.108
Alistipes	0.482	0.0297	1.05	0.21	0.0344	0.507	0.355
Odoribacter	0.0416	0	0.0815	0	0	0.0149	0.033*
Clostridiales	2.92	1.23	4.6	3.07	2.19	6.21	0.231
Christensenellaceae	0.0844	0.0129	0.266	0.365	0.0421	0.942	0.172
Clostridiaceae	0.0188	0.00147	0.0916	0.0545	0.0248	0.244	0.06
Clostridium	0.11	0.034	0.258	0.423	0.0865	1.66	0.047*
Oscillospira	1.08	0.122	1.76	0.911	0.24	3.02	0.55
